# Identification of Specific microRNAs in Adipose Tissue Affected by Lipedema

**DOI:** 10.3390/cimb46110710

**Published:** 2024-10-25

**Authors:** Erika Cione, Sandro Michelini, Diana Marisol Abrego-Guandique, Nicola Vaia, Serena Michelini, Valeria Puleo, Matteo Bertelli, Maria Cristina Caroleo, Roberto Cannataro

**Affiliations:** 1 GalaScreen Laboratories, Department of Pharmacy, Health and Nutritional Sciences, University of Calabria, 87036 Rende, Italy; rcannataro@nutrics.it; 2 Servizio di Diagnostica e Riabilitazione Vascolare Ospedale di Marino, 00047 Rome, Italy; sandromichelini2018@gmail.com; 3 Department of Health Sciences, University of Magna Graecia Catanzaro, 88100 Catanzaro, Italy; dianamarisol.abregoguandique@unicz.it (D.M.A.-G.); mariacristina.caroleo@unicz.it (M.C.C.); 4 Chirurgia Plastica, Ricostruttiva ed Estetica, European Hospital, 00149 Rome, Italy; nicola.vaia@gmail.com; 5 Medicina Fisica e Riabilitazione, Università La Sapienza, Ospedale S. Andrea, 00185 Rome, Italy; serenamichelini@gmail.com; 6 Dipartimento di Scienze e Sanità Pubblica, Università Cattolica Policlinico Gemelli, 00168 Rome, Italy; puleova@gmail.com; 7 MAGI Euregio, 39100 Bolzano, Italy; bertellimatteo@hotmail.com; 8 Research Division, Dynamical Business and Science Society—DBSS International SAS, Bogotá 110311, Colombia

**Keywords:** lipedema, microarray, microRNAs, epigenetic

## Abstract

Lipedema is a chronic disorder affecting women with a 10% incidence worldwide. It is often confused with obesity. This study was undertaken to study microRNAs in lipedema tissue assessed by direct hybridization using the robust n-counter flex DX CE-IVD platform. The mean age of the subjects participating in the study was 40.29 (±12.17). The mean body weight and BMI were 67.37 (±10.02) and 25.75 (±4.10), respectively. The lipedema stages included were I and II. The differential expressed human (hsa)-miRNAs were determined according to a log2 fold-change (LFC) of 0.5 and *p* value < 0.05. To these, increased expression of hsa-let-7g-5p was evident, as well as reduced levels of hsa-miR-371a-5p, -4454+7975, -365a+b-3p, -205-5p, -196a-5p, -4488, -2116-5p, -141-3p, -208a-3p, -302b-3p, 374a-5p, and -1297. Then, several bioinformatics tools were used to analyze microarray data focusing on validated target genes in silico. KEGG and Gene Ontology (GO) pathway enrichment analysis was conducted. Furthermore, the protein–protein interaction and co-expression network were analyzed using STRING and Cytoscape, respectively. The most upregulated miRNA mainly affected genes related to cell cycle, oocyte meiosis, and inflammatory bowel disease. The downregulated microRNAs were related to endocrine resistance, insulin resistance, hypersensitivity to AGE-RAGEs, and focal adhesion. Finally, we validated by RT-PCR the upregulated hsa-let-7g-5p and two down-regulated ones, hsa-miR-205-5p and hsa-miR-302b-3p, confirming microarray results. In addition, three mRNA target miRNAs were monitored, SMAD2, the target of the hsa-let-7g-5p, and ESR1 and VEGFA, the target of hsa-miR-205-5p and hsa-miR-302b-3p, respectively. Our results open a new direction for comprehending biochemical mechanisms related with the pathogenesis of lipedema, shedding light on this intricate pathophysiological condition that could bring to light possible biomarkers in the future.

## 1. Introduction

Defined for the first time by Hines and Allen in 1940, lipedema is a chronic pathology still not well identified and, therefore, difficult to diagnose [1,2]. The disfunction is classically present in females and is confused, often, with lipodystrophy, lymphoedema, or obesity. It has an estimated incidence of about 10% worldwide [3,4]. Five types of lipedema are classified today based on its anatomical location and into three stages dependent on its severity [5]. Briefly, stage 1: skin presents a smooth texture with a subdermal pebble-like feel due to underlying loose connective tissue fibrosis, and the patients often have a normal weight; stage 2: fatty deposits appear on the skin due to the progressed fibrotic changes and excess tissue, with palpable nodules; and stage 3: the skin features increased edema in adipose tissue that is more fibrotic in texture, with numerous large subdermal nodules and overhanging lobules of tissue. The patients in these two latter stages are often overweight/obese. There is currently no therapy to treat lipedema, even if nutritional supplements could be used to manage edema [6]. The ketogenic diet achieved a successful nutritional approach [7,8,9,10,11]. The diagnosis is clinical; no marker can characterize the condition, which seems to depend on the hormonal state. The onset is very often related to menarche, but variations are also noted in pregnancy, especially if assisted by hormonal therapies and menopause [12,13]. Recently, mutations of genes associated with non-syndromic lipedema [14] and soluble factors linked to lymphatic vasculature dysfunction, which in turn is an important component in the pathogenesis of lipedema, were discovered [15]. However, these discoveries, genotypes, and phenotypes are not always linked, especially in complex and multifaceted diseases [16] as it is also lipedema. The way in which the environment, via epigenetic biochemical mechanisms, can affect health status is known [17,18]. In this context, microRNAs (miRNAs), small non-coding RNAs, are powerful epigenetic regulators or potential biomarkers [17,18,19]. The present study aimed to identify specific miRNAs in the adipose tissue affected by lipedema, also exploring the relationship between miRNA–mRNA interactions. Thus, we compared miRNA expression profiles in lipedema subcutaneous adipose tissue (SAT) leg biopsies with biopsies from a subscapular area from the same participants. We performed a microarray using the n-counter flex DX CE-IVD platform to identify differential expression (DE) of miRNAs and analyzed the data using bioinformatics-based approaches. It is worth mentioning here that the n-counter DX CE-IVD platform and qPCR, in terms of analytical performance, provided similar results, with the advantage of the n-counter platform meritted to the fact that it is quicker, more accessible to multiplex, and almost fully automated [20]. In any case, we used RT-PCR methodology to validate the specific miRNAs obtained by the array and targets.

## 2. Materials and Methods

### 2.1. Subjects and Study Design

The study protocols were approved by Azienda Sanitaria dell’ Alto Adige, Italy (Nr 130-2021, accepted on 18 October 2021). All participants fulfilled the written informed consent. The study was conducted according to the principles of the Declaration of Helsinki. The study was composed of 12 patients affected by lipedema. The sample for this study was derived from Vascular Diagnostics and Rehabilitation Service ASL Roma 6, Marino (RM), director Sandro Michelini. We included patients with lipedema defined using the well-established histopathologic criteria in the present study. The study included pathological (lipedema) tissue derived from biopsy subcutaneous adipose tissue (SAT) from the legs and from a non-affected subscapular area (considered as healthy tissues) from the same participants. The samples were obtained by 12 participants, and there were 24 samples in total. All the samples were collected between 2021 and 2022 through a fat pad biopsy with 10% neutral buffered formalin and stored at −80 °C until the analysis. Then, tissues were defrosted, embedded in optimal cutting temperature (OCT) solution, and then sectioned into 50 µm thick tissues with a microtome/cryostat.

### 2.2. Direct miRNA Hybridization Array

The total RNAs were extracted using miRNeasy tissue/cells advanced Mini Kit purchased from Qiagen (Hilden, Germany) according to the manufacturer’s instructions.

Adipose tissue RNA extraction can be challenging due to high lipid content and low cell numbers [21]. From the samples, we were able to extract and analyze 6 samples of lipedema and 5 of the corresponding healthy tissues since the RNA was not enough for one. Since the nanostring cartridge allocates 12 samples, we included 1 extra lipedema sample. The array was conducted on the n-counter DX CE-IVD certified platform of NanoString Technology (Seattle, WA, USA). A total of 100 ng of RNA/miRNA was used as an input. Mature miRNAs underwent to annealing procedure. Then, 1 μL of Ligase was added directly to the bottom of each tube. The ligation protocol started to have a species-specific sequence. After finishing the ligation protocol, the un-ligated miRtags were removed by enzymatic purification through addition of the 1 μL ligation clean-up enzyme to each tube to initiate the purification protocol following thermal cycler conditions of 37 °C for 60 min, followed by 70 °C for 10 min. Then, miRtagged mature miRNAs were hybridized with an nCounter Human (V3) miRNA Expression Assay CodeSet following the manufacturer’s instruction (code CSO-MIR3-12). The hybridization reaction was performed for 22 h. The n-counter DX is composed of the nCounter Prep Station, in which the unhybridized CodeSet was removed by automated purification and the nCounter Digital Analyzer, where the remaining target probes’ complexes are counted by scanning at 555 fields of view (FOV). The data output was imported into nSolver™ (NanoString Technologies, Seattle, WA, USA). Then, raw excel table data were exported for further bioinformatics analysis. The same approach was used by our group previously [22,23,24].

### 2.3. Real Time-PCR microRNAs Validation

In order to validate the microRNA expression derivates by array, real-time PCR was per-formed with QuantStudio™ 5 system (PCR System, Applied Biosystems™, Madrid, Spain). The cDNA template was prepared using the TaqMan Advanced miRNA cDNA Kit (Thermo Fisher Scientific, Waltham, MA, USA). The isolated RNAs were used as input for a total of 2 µL (5 ng/µL). Following the TaqMan Advanced miRNA user guide, the reaction products obtained were used in the subsequent adaptor ligation, reverse transcription (cDNA) synthesis reactions, and then the miR-Amp reaction was performed. The following cycling conditions were used: 95 °C for 20 s, 40 cycles at 95 °C for 1 s, and 60 °C for 20 s. The PCR reactions were set up using 5 µL of the diluted cDNA (1:10 with RNAse-free water) with 10 µL of 2X TaqMan Advanced Master, 4 µL of RNase-free water, and 1 µL of 20X TaqMan Advanced miRNA Assay containing the primers. The RT-PCR analyses were carried out using the TaqMan probe for the miRNA hsa-let-7g-5p 478580_mir (UGAGGUAGUAGUUUGUACAGUU) and the TaqMan probes for miRNAs hsa-miR-302b-3p 478591_mir (UAAGUG-CUUCCAUGUUUUAGUAG) and hsa-miR-205-5p 477967_mir (UCCUUCAUUCCAC-CGGAGUCUG). RNU48 was used as an internal control [25] to calculate the relative expression of microRNAs using the 2^–ΔΔCt^ method.

### 2.4. Real Time-PCR mRNAs Monitoring

Candidate target mRNAs were also confirmed by RT-PCR. The total RNA isolated was converted to cDNA by use of the e iScript cDNA Synthesis kit by Bio-Rad (Hercules, CA, USA), which was followed by real-time PCR. Gene expression was determined by running 5 µL of cDNA template to a final volume of 20 µL on a QuantStudio™ 5 Real-Time PCR System (Applied Biosystems™, Madrid, Spain) using a TaqMan Fast Advanced Master Mix (Thermo Fisher Scientific; Waltham, MA, USA). Thermal cycling conditions were: 20 s at 95 °C for initial denaturation, followed by 40 cycles with 1 s denaturation at 95 °C and 20 s annealing and elongation at 60 °C. Fold of change in mRNA was calculated by the 2^–ΔΔCt^ method and expression levels of gene Smad Family Member 2 (*SMAD2*) (Assay ID: Hs00998187_m1), Estrogen Receptor 1 (*ESR1*) (Assay ID: Hs01046816_m1), and Vascular endothelial growth factor A (*VEGFA*) (Assay ID: Hs00900055_m1) were normalized to the tissue-appropriate reference gene, 18S (Assay ID: Hs03003631_g1). All probes were purchased from Thermo Fisher Scientific (ThermoFisher Scientific, Waltham, MA, USA). Compliance with the Minimum Information for the Publication of Real-Time Quantitative PCR Experiments (MIQE) guidelines [26] was provided, and all experiments were performed in triplicate.

### 2.5. Identification of Target Genes

Target genes were identified based on the miRWalk algorithm, which uses the three databases miRWalk, miRDB, and TargetScan to asses miRNA–target interactions only on validated target genes. These data were further confirmed by the miRTarbase platform.

### 2.6. Gene Ontology and KEGG Pathway Enrichment Analysis

Gene ontology (GO) and KEGG analysis of the validated targets of the co-differentially expressed miRNAs in lipedema and healthy tissue were performed. ClusterProfiler R package 4.2.1 was used to perform GO enrichment analysis to annotate and infer the functions of these miRNAs. The plugin “BiNGO” tool in the “Apps” of the Cytoscape was used for the GO enrichment. As a result, the network of GO enriched the genes into 3 terms: Molecular Function (MF), Biological Process (BP), and Cellular Component (CC) were created. Moreover, GO enrichment analysis parameters of BP, CC, and MF were obtained from http://geneontology.org/ (accessed on 5 November 2023) and dotplot created by SRPLOT tools [27].

### 2.7. Protein–Protein Interaction Networks

The interaction networks at the gene level were built by the GeneMANIA Cytoscape plugin. Gene co-expression networks of key genes were constructed and Physical, co-expression, and pathway gene–gene interactions were evaluated. The STRING online tool was used to identify protein–protein interactions of identified key genes [28]. It provides direct (physical) interactions and indirect (functional) associations; they stem from computational algorithms, knowledge transfers between organisms, and interactions aggregated from other (primary) databases. Moreover, targeted genes were clustered using STRING. Subsequently, a sub-network was built using the MCODE Cytoscape plugin [29].

### 2.8. Statistical Analysis

For data normalization, the DESeq2 R package was used [30]. Statistically significant DE miRNAs were defined as LFC 0.5, and *p* < 0.05 were used as cut-offs to filter DE miRNAs. Volcano plots were created using SRPLOT [27]. The heatmap was created using R pheatmap (v1.0.12). All the packages used in R software 4.2.1 were downloaded from Bioconductor [31]. Similarly, a cut-off of *p* < 0.05 was used for significant enrichment results in GO and KEGG analyses. To validate miRs and targets, an independent t-test was performed to compare miRNA and gene expression between the two groups, with statistical significance set at *p* < 0.05. The results were visualized using GraphPad Prism version 10.2.3 for Windows (GraphPad Software, La Jolla, CA, USA).

## 3. Results

### 3.1. Characteristics of the Participants

Participants’ sociodemographic and anthropometric characteristics are listed in Table 1. The mean age for the subjects as a whole group was 40.29 (±12.17) years old. The mean body weight and BMI were 67.37 (±10.02) and 25.75 (±4.10), respectively. The most common lipedema type was stage 2, reported among 66.6% of participants. Additionally, 83.3% of the participants in this sample had experienced pain; all participants presented edema, while 75% presented paraesthesia and dysesthesia. Finally, 50% of the patients presented hematoma and 25% ecchymosis.

### 3.2. Nanostring Analysis and Differentially Expressed miRNAs

**Figure 1:**
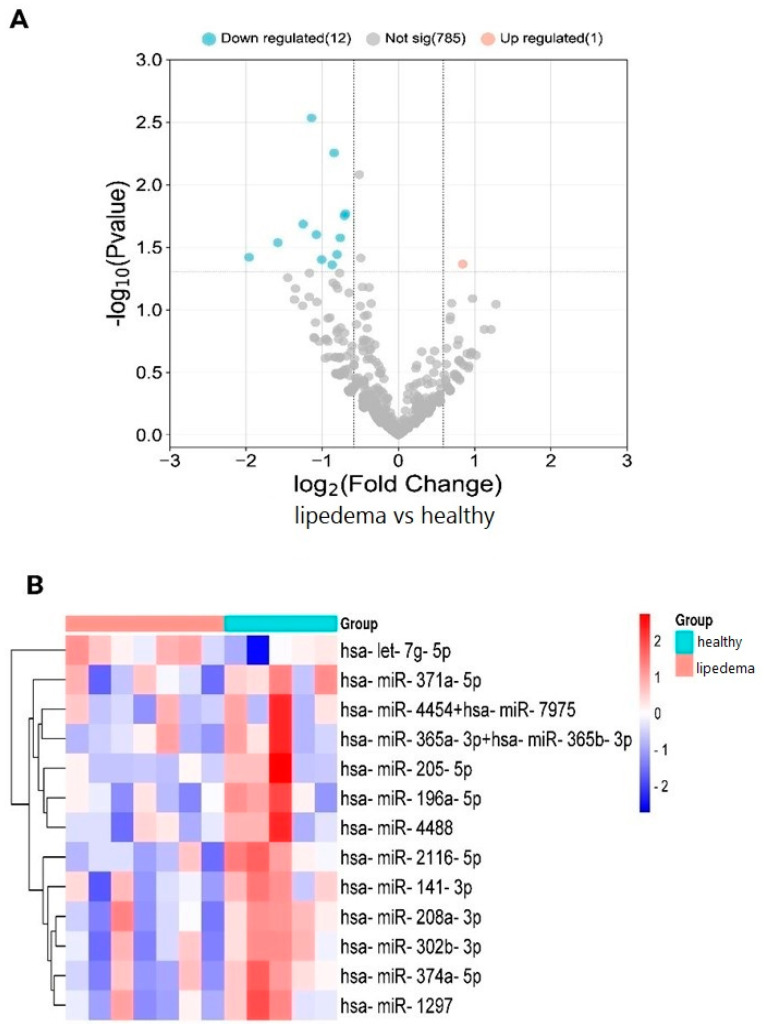
(**A**) Volcano plot of lipedema versus healthy tissue. Blue spots represent downregulated miRNAs (hsa-miR-371a-5p, -4454+7975, -365a+b-3p, -205-5p, -196a-5p, -4488, -2116-5p, -141-3p, -208a-3p, -302b-3p, 374a-5p, and -1297), and the pink spot represent the upregulated miRNA (hsa-let-7g-5p). Gray spots represent miRNAs that did not show significant changes between the two groups. (**B**) Heatmap of differentially expressed miRNAs (*n* = 7 of lipedema tissue; *n* = 5 of healthy tissue).

The nucleic acid concentration of total RNA was isolated from two adipose tissue areas of the same subject. Only 12 tissue samples of the total 24 (7 lipedema and 5 healthy) had sufficient quality and quantity of total RNAs for subsequent analysis; therefore, 7 samples of leg lipedema (4 samples in stage I and 3 samples in stage II) and 5 samples from the subscapular area were indicated as healthy (4 samples in stage I and 1 sample in stage II). The spike-in and positive control expression levels were similar in all the samples that showed respectable extraction and ligation. Hybridization was efficient in all the tissue samples during sample preparation. The nCounter miRNA expression profiling panel simultaneously detects > 700 miRNAs. The Volcano plot based on the most differential expression (DE) of miRNAs is shown in Figure 1A. Of these, 1 resulted upregulated, and 12 miRNAs were downregulated. The heatmap and hierarchical clustering based on the most DE miRNAs are shown in Figure 1B. Among these, the hsa-let-7g-5p was the only one upregulated, and hsa-miR-371a-5p, -4454+7975, -365a+b-3p, -205-5p, -196a-5p, -4488, -2116-5p, -141-3p, -208a-3p, -302b-3p, 374a-5p, and -1297, were downregulated. Figure 1B shows a dendrogram combined with a heatmap to show the clustering of miRNAs that are similarly expressed.

### 3.3. Target of Differentially Expressed miRNAs

There are 80 unique targets of upregulated miRNAs, 170 unique targets of downregulated miRNAs, and 13 targets shared between both groups. The overlapping genes found between the upregulated and downregulated miRNAs are LRIG3, RDX, TGFBR3, PPP1R15B, YOD1, ZNF644, MARS2, IGF1R, HMGA1, MAP3K1, IGF2BP1, NAP1L1, and SMCR8. Table 2 integrates all miRNAs with their targets. The miRNAs are divided into upregulated and downregulated (details in Supplementary Materials Tables S1 and S2). The only upregulated miRNA targets a wide range of genes. These genes are associated with cell growth (e.g., IGF1R), apoptosis (e.g., BCL2L1), signaling pathways (e.g., MAP3K1, SMAD2, IL13, and TGFBR3), and other cellular functions. Among the downregulated miRNAs, hsa-miR-371a-5p targets a limited number of genes, including MYCN (an oncogene) and SOX2 (involved in stem cell maintenance); while hsa-miR-365a-3p affects genes involved in apoptosis (e.g., BCL2), hsa-miR-205-5p instead regulates genes related to cell adhesion (e.g., VEGFA) and the transcription factors ZEB1 and ZEB2. The targets of hsa-miR-196a-5p play roles in development, growth, and RNA processing (e.g., HOXA7, HOXC8, IGF2BP1, IGF2BP3). The miRNA hsa-miR-141-3p influences genes involved in cell proliferation, apoptosis (e.g., PTEN), and transcription regulation (GATA6, HOXB5, and KLF12). In addition, hsa-miR-208a-3p regulates ETS1 and MED13, which are linked to transcription regulation. Finally, hsa-miR-302b-3p has a wide range of target genes, including those involved in the cell cycle (e.g., CCND2, CDK2), transcription factors (e.g., IRF2, KLF3), and hormone signaling (e.g., ESR1).

### 3.4. Gene Ontology (GO) Analysis of the Differentially Expressed miRNAs Target Genes

**Figure 2:**
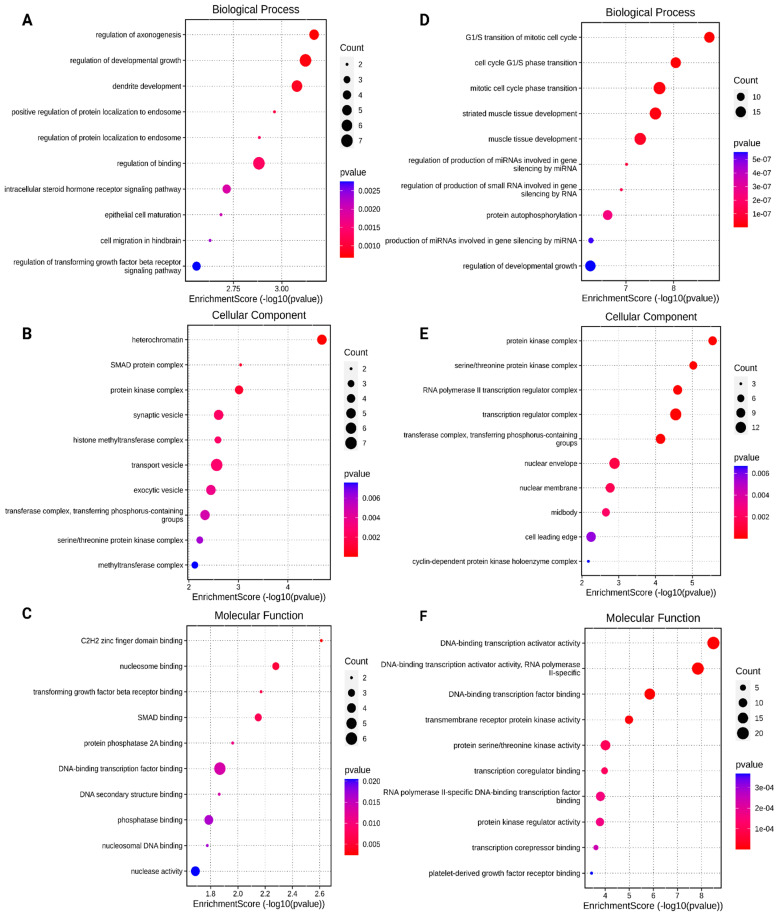
Gene Ontology analysis of target genes of miRNAs differentially expressed. (**A**–**C**) (left) correspond to the upregulated miRNA targets, and (**D**–**F**) (right) to the downregulated miRNA targets. The panels show the top 10 most represented Biological Processes (**A**,**D**), Cellular Components (**B**,**E**), and Molecular Functions (**C**,**F**) among the DE miRNA targets. The color of each dot represents the *p* value of each term involved in the analysis. The size of each dot represents the counts of overlapped genes between the input genes and the total gene list on the GO pathway.

To understand the biochemical functions and connections of the DE miRNAs in lipedema tissue, enrichment analyses were applied to clarify the biological function of miRNAs signature through target genes in Gene Ontology (GO) analysis [32]. Genes with a significant enrichment statistic, assessed by each GO, and linked to biological processes (BP), cellular components (CC), and molecular functions (MF), were considered. The results are shown in Figure 2 of upregulated and downregulated miRNAs, respectively. The only miRNA upregulated, hsa-let-7g-5p, resulted in a GO Three ontology associated with BP (Figure 2A) linked to regulation of axogenesis, developmental growth, dendrite development, positive regulation of protein localization to endosomes, regulation of binding, intracellular steroid hormone receptor signaling pathways, epithelial cell maturation, cell migration in the hindbrain, and transforming growth factor β receptor signaling pathways. Additionally, CC were enriched in heterochromatin, including the SMAD protein complex, protein kinase complex, synaptic vesicles, the histone methyltransferase complex, transport and exocytic vesicles, methyltransferase for phosphorus-containing groups, the serine/threonine complex, and the methyltransferase complex (Figure 2 panel B). Among the top 10 pathways represented by MF, C2H2 zing finger domain binding, nucleosome binding, transforming growth factor β receptor factor, SMAD binding, protein phosphatase 2A binding, DNA binding transcription factor, DNA secondary structure, phosphatase binding, nucleosome DNA binding, and nuclease activity were found (Figure 2 panel C). For the downregulated miRNAs, GO Three ontology associated miRNAs with BP (Figure 2D) including the G1/S transition of mitotic cell cycle, cell cycle G1/S phase transition, mitotic cell cycle phase transition, striated muscle tissue development, muscle tissue development, regulation of production of miRNAs involved in gene silencing by RNA, regulation of production of small RNA involved in gene silencing by RNA, protein autophosphorylation, production of miRNAs involved in gene silencing by RNA, and regulation of developmental growth. Furthermore, the CC (Figure 2E) were enriched in the protein kinase complex, serine/threonine protein kinase complex, RNA polymerase II transcription regulator complex, transferase complex, transferring phosphorus-containing groups, nuclear envelope, nuclear membrane, midbody and cyclin-dependent protein kinase holoenzyme complex. Finally, the MF (Figure 2F) pointed out included DNA-binding transcription activator activity, DNA-binding transcription activator activity that is RNA polymerase II-specific, DNA-binding transcription factor binding, transmembrane receptor protein kinase activity, protein serine/threonine kinase activity, transcription coregulator binding, RNA polymerase II-specific DNA binding transcription factor binding, protein kinase regulator activity, transcription corepressor binding and platelet-derived growth factor receptor binding.

### 3.5. KEGG Pathway Functional Enrichment Analysis

**Figure 3:**
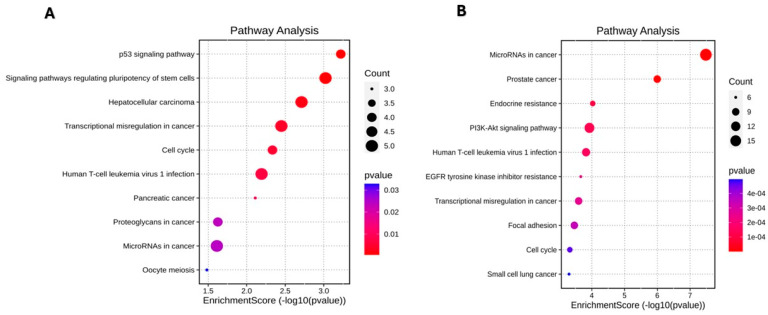
KEGG pathways analysis of target genes by miRNAs. (**A**) upregulated and (**B**) downregulated miRNAs of inflamed vs. healthy tissue. The color of each dot represents the *p* value of each term involved in the analysis. The size of each dot represents the counts of overlapped genes between the input genes and the total gene list on the KEGG pathway.

KEGG consists of databases with information about genomes, biological pathways, diseases, drugs, and chemical substances [33]. The top 10 pathways enriched by the target genes are also displayed in the dot plot shown in Figure 3A,B, it did not report enrichment linked to cancer. In the top 10 enriched KEGG pathways of upregulated miRNA, the p53 signaling pathways, signaling pathways regulating pluripotency of stem cells, cell cycle, and oocyte meiosis have been shown as prominent (Figure 3A), while for the downregulated miRNAs, preeminence was on endocrine resistance, PIK3/Akt signaling pathway, EGFR tyrosine kinase inhibitor resistance, focal adhesion, and the cell cycle (Figure 3B).

In addition, it was identified that some KEGG pathways and genes that upregulated miRNA might regulate and participate in the pathogenesis of lipedema (Table 3), such as genes linked to cell cycle, oocyte meiosis, and inflammatory bowel disease, while KEGG pathways through downregulated miRNAs are linked to endocrine resistance, EGFR tyrosine kinase inhibitor resistance, focal adhesion, the cell cycle, the TGF-β signaling pathway, the Hippo signaling pathway, the FoxO signaling pathway, the AGE-RAGE signaling pathway in diabetic complications, Cushing syndrome, insulin resistance, the HIF-1 signaling pathway, the prolactin signaling pathway, and the AMPK signaling pathway. Details of KEGG pathways are present in the Supplementary Materials.

### 3.6. Protein–Protein Interaction Network Analysis

**Figure 4:**
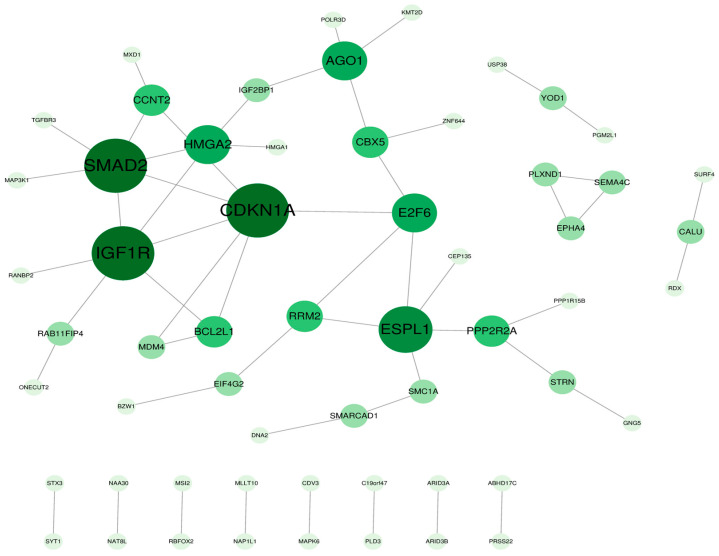
Protein–protein interaction network. Each node is a protein, and an edge is an interaction between two proteins. The green circle indicates the protein, and the node size represents the degree of value. The node size represents the degree value.

Interactions among these genes resulting in a translated protein were explored. The protein–protein interaction (PPI) was applied, and the most important modules were then screened. Two PPI networks were created; the only upregulated miRNA with 58 nodes and 54 edges was obtained, and the average number of neighbors was 1.86. The hub nodes with a high number of neighbors (≥6), such as CDKN1A, IGF1R, and SMAD2, were identified (Figure 4). The Molecular Complex Detection (MCODE) did not generate sub-network significance.

**Figure 5:**
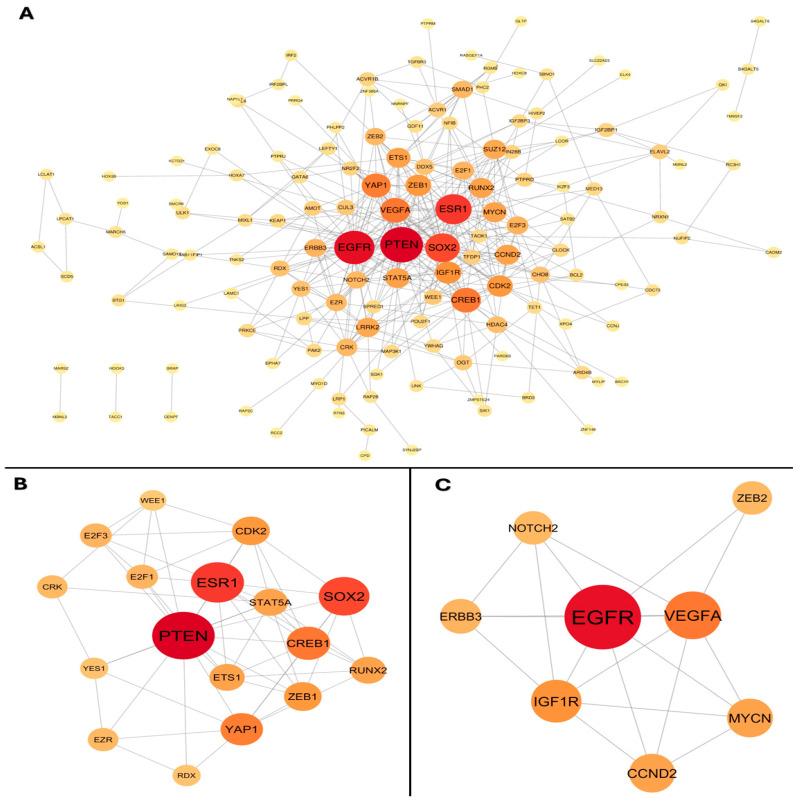
Network and Sub-network Protein–protein interaction of downregulated miRNAs. (**A**) Whole network protein–protein interaction. (**B**) Sub-network PTEN, ESR1, and SOX2. (**C**) Sub-network EGFR, VEGFA, and IGF1R. Each node is a protein, and an edge is an interaction between two proteins. The node size represents the degree value.

The second network was obtained by the downregulated miRNAs with a count of 142 nodes and 405 edges, and the average number of neighbors was 5.70. The hub nodes with the most significant number of neighbors (≥20), including PTEN, EGFR, ESR1, SOX2, CREB1, VEGFA, and YAP1 (Figure 5A). This latter network, by MCODE, has two sub-networks that were constructed; the first with a score of 7.12 was obtained, including 17 nodes such as PTEN, ESR1, and SOX2. (Figure 5B) and the second module with a score of 5.43 and 8 nodes and 19 edges, including EGFR, VEGFA, and IGF1R (Figure 5C). 

### 3.7. Validation by RT-PCR of Differentially Expressed microRNAs and Monitoring of mRNA Targets

**Figure 6:**
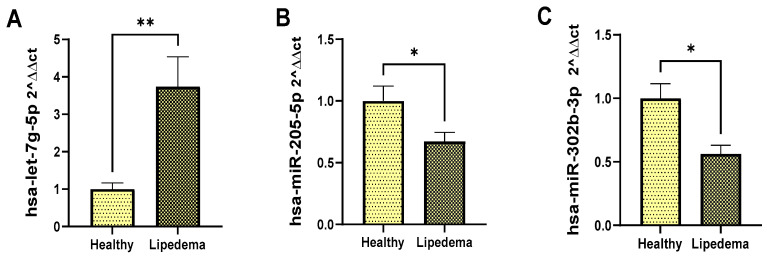
**RT-PCR analysis of miRNAs expression**. Relative mRNA levels in lipedema compared to healthy tissue: (**A**) hsa-let-7g-5p, (**B**) hsa-miR-205-5p, (**C**) hsa-miR-302b-3p. MiRNAs are normalized to the RNU48 RNA of each sample. Statistical analysis with *test-t* was as follows: * *p* < 0.05; **, *p* ≤ 0.001. Data are mean ± SEM (*n* = 7 of lipedema tissue; *n* = 5 of healthy tissue).

Representative microRNAs were validated by the RT-PCR platform; the only miRNA upregulated was hsa-let-7g-5p (Figure 6 panel A), and two downregulated miRNAs, hsa-miR-205-5p (Figure 6 panel B) and hsa-miR-302b-3p (Figure 6 panel C), are shown. The RT-PCR results display a similar direction of expression change to the observed microarray results, with significant results when comparing healthy versus the lipedema group.

**Figure 7:**
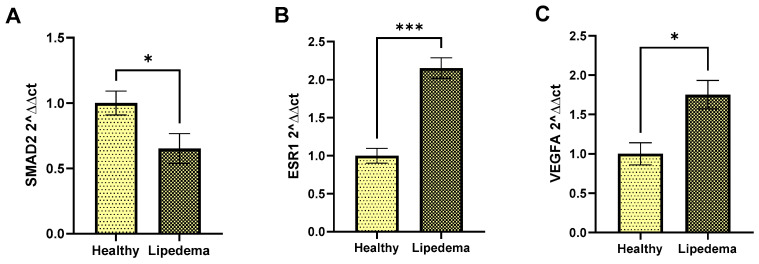
**RT-PCR analysis of mRNAs**. Relative expression of mRNA levels in lipedema compared to healthy tissue: (**A**) SMAD2, (**B**) ESR1, (**C**) VEGFA; miRNAs are normalized to 18S RNA of each sample. Statistical analysis with *t*-test was as follows: * *p* < 0.05; *** *p* ≤ 0.0001. Data are mean ± SEM (*n* = 7 of lipedema tissue; *n* = 5 of healthy tissue).

Based on the KEGG enrichment analyses and PPI expression data. Three mRNA targets of the validated miRNAs above were monitored through RT-PCR, including SMAD2, the target of hsa-let-7g-5p, (Figure 7A), ESR1, the target of hsa-miR-302b-3p (Figure 7B), and VEGFA, the target of hsa-miR-205-5p (Figure 7C).

## 4. Discussion

Our scientific approach in performing the direct hybridization array led us to identify different expression (DE) miRNAs in lipedema tissue compared to healthy tissues. Lipedema displayed DE of thirteen miRNAs, one upregulated and twelve downregulated. With the methodological approach used here, we identified tissue miRNAs reflecting the pathogenesis of lipedema adipose tissue. The microRNAs identifyed here include hsa-let-7g-5p (upregulated), and hsa-miR-371a-5p, -4454+7975, -365a+b-3p, -205-5p, -196a-5p, -4488, -2116-5p, -141-3p, -208a-3p, -302b-3p, 374a-5p, and -1297 (downregulated). In addition, bioinformatic data analysis on validated target genes indicates the functions of these DE miRNAs by GO and KEGG analysis. With this knowledge, our findings provide an overall vision of miRNAs in lipedema pathophysiology, unveiling parallels with the clinical observations of this condition. In this regard, estrogen has been postulated to play a significant role in its pathophysiology. Estrogen, may contribute to the development of lipedema since it is a key regulator of adipocyte glucose, lipid metabolism [34], and female-associated body fat distribution [35]. Studies have proposed that lipedema is almost exclusively observed in females, indicating a potential estrogen requirement for its manifestation; it commonly appears at puberty and during pregnancy [36]. The KEGG pathway for the upregulated miRNA highlighted the involvement of cell cycle and oocyte meiosis biochemical pathways. Furthermore, impairments in the activity of estrogen or the absence of estrogen receptors have been linked to the subcutaneous adiposity accumulation, a characteristic of lipedema [37]. Additionally, hormonal changes, including increased estrogen levels or reduced testosterone levels, have been associated with lipedema in males affected [38]. In our study, GO analysis showed that targets of DE miRNAs in lipedema tissue were enriched in intracellular steroid hormone receptor signaling pathway changes, whereas KEGG pathways highlighted endocrine resistance, including insulin and prolactin signaling pathways to act through loss or modification of estrogen receptor activity; it has been hypothesized that impaired estrogen receptor expression and signaling may be involved in the onset of lipedema, as estrogen affects lipid metabolism directly, especially in white adipose tissue via its receptor [39]. In addition, estrogen controls the vascular endothelial growth factor-A (VEGFA) in adipose tissue. Siems et al., demonstarted that VEGFA was found to be increased in lipedema patients, resulting in an increased capillary fragility and pathological angiogenesis [40]. Lipedema is characterized by higher endothelial permeability and altered endothelial cell junctions. Changes in endothelial permeability were reported to be associated with the redistribution of cell surface proteins and stabilization of focal adhesion according to our KEGG pathway analysis [41], whereas in another study the levels of VEGFA remained comparable to the control subjects [42]. Moreover, the impairment of estrogen signaling may also be a culprit in the lymphatic dysfunction in lipedema [41], although the presence of a lymphatic vascular component is still controversial. Another common characteristic of lipedema adipocytes seems to be hyperproliferation due to excessive mitotic clonal expansion [43]. Some targets of our miRNAs, such as IGF-1 signaling through the AMPK signaling pathway, promote mitotic clonal expansion in the initial stages of adipogenesis [44]. At the same time, the CCND2 gene was found to be associated with adipose tissue development and differentiation [45]. Moreover, it has been reported that KLF is associated with preadipocyte differentiation and lipid accumulation [46]. Adipogenesis or adipocyte differentiation is regulated by Tyrosin Kinases [47]. Adipose tissue-derived stem cells are regulated by receptor tyrosine kinases, including ERBB receptors and the downstream-regulated serine/threonine protein kinase B and PTEN activity [48]. In the context of lipedema, elevated M2 macrophages, lymphocyte subtypes, and PF4—an inflammatory marker—have been associated with the condition, indicating a potential link between inflammation and the pathogenesis of lipedema [3]. Ma et al., demonstrated elevated levels of PF4 in circulating blood plasma exosomes from lipedema patients [15], permitting to discriminate lipedema and lymphedema from obesity. Moreover, ketogenic diet shows antioxidant profile [49] and was effective [7] not in the later stages adiposity disfunction, where there is an increase in sodium levels, which in turn limits the lymphatic clearance. Also, elevated sodium levels may mainly attract salt-sensing macrophages, releasing inflammatory mediators in lipedema [50]. Elevated tissue sodium may be correlated to lower extremity pain in patients with lipedema [51]. Indeed, pain is the leading symptom of lipedema [52]. In our study, pathways related to inflammation, such as the FoxO signaling pathway, AGE-RAGE signaling pathway, and TGF-β signaling pathway, affected DE miRNAs, such as dysregulation of hsa-let-7g-5p related to chronic adipose tissue inflammation [53]. For instance, Choo et al. demonstrated the involvement of hsa-let-7g-5p in targeting IGF1R, which regulates angiogenesis and apoptosis [54]. Several regulatory pathways mediate the effects of hsa-let-7g. Yi et al. proved that let-7g directly regulated TGFβR and SMAD2 in TGF-β signaling present in our bioinformatic approach [55]. In this view, we validated in RT-PCR the expression of hsa-let-7g-5p (upregulated), hsa-miR-205-5p, and hsa-miR-302b-3p (both downregulated), confirming the result obtained in the microarray analysis. Those microRNAs target the mRNAs of SMAD2, ESR1, and VEGFA, which were also monitored. Thielen et al. evidence that inflammation inhibits SMAD2 in some conditions [56]. Furthermore, SMAD2 conditional knockout mice did not exhibit significant effects on weight gain, irrespective of diet [57]. In addition, our results evidence the upregulation of ESR1 and VEGFA mRNAs targets of hsa-miR-205-5p and hsa-miR-302b-3p, respectively. Activation of ESR1 increased VEGFA expression, which changes adipose tissue deposits [58]. On the other hand, VEGFA, a lymphatic-related cytokine was among the inflammatory markers, upregulated in lipedema, and has also been related to lipedema progression [59]. Here, we analyzed for the first time miRNAs in the lipedema tissue and identified them as possible candidate biomarkers for this condition. It is worth noting that miRNAs circulate in the plasma/serum mainly in exosome vesicles secreted in a tissue-specific manner [60,61,62], and specific miRNA methylation is a diagnostic marker for human papillomavirus [63]. Therefore, our next steps will evaluate the microRNAs identified here in blood serum since extracellular vesicles derived from the stromal vascular fraction of adipose tissue were found to carry characteristic miRNAs, which were significantly increased in lipedema [48,53,64]. The identification of specific miRNAs in the context of lipedema was driven by the need to better understand the molecular mechanisms underlying the disease. Dysregulated miRNAs can play a crucial role in the regulation of key biological pathways associated with adipose tissue dysfunction and inflammation, which are central to lipedema pathology.

To the best our knowledge, this is the most extensive miRNA profiling analysis completed in the adipose tissue of lipedema compared to healthy adipose tissue. This study presents a homogenous patient group; the data were obtained by two biopsies from the same person to overcome the intrinsic biological diversity from person to person. It is worth mentioning here that the n-counter DX CE-IVD platform and qPCR, in terms of analytical performance, provided similar results, even in our case, with the advantage of n-counter being that it is quicker, almost fully automated [60], and reliable for daily clinical practice, besides its use in validating RNA-sequencing data [19,65]. While our study provides valuable insights into the role of miRNAs and their target mRNAs in lipedema, some limitations should be considered: (i) small sample size and lack of stage-specific analysis, due to the limited number of samples in stage; (ii) technical challenges in adipose tissue RNA extraction. Our study focused exclusively on miRNA and mRNA expression in adipose tissue, which may not capture the full complexity of lipedema pathology.

## 5. Conclusions

In conclusion, our results open a new direction for understanding the biochemical mechanisms associated with the pathogenesis of lipedema, shedding light on this intricate pathophysiological condition. Furthermore, since microRNAs from tissue are usually released in blood circulation, the microRNAs identified here could be designed as possible biomarkers for lipedema.

## Figures and Tables

**Table 1:** Participants characteristics.

Variable	*n* = 12 (±SD)
Age	40.29 (±12.17)
Weight (Kg)	67.37 (±10.02)
BMI	25.75 (±4.10)
Stage	
I	4 (33.3%)
II	8 (66.6%)
Physical characteristics	
Pain	10 (83.3%)
Edema	12 (100%)
Paraesthesia	9 (75%)
Dysesthesia	9 (75%)
Injury	
No	5 (41.6%)
Hematoma	6 (50%)
Ecchymosis	3 (25%)

The results are present as mean ± standard deviation or percentage.

**Table 2:** Target genes of differential miRNAs expression in lipedema.

miRNA Upregulated	mRNA Targets
hsa-let-7g-5p	ABHD17C, ACER2, AGO1, AP1S1, ARID3A, ARID3B, BCL2L1, BEND4, BZW1, C19orf47, C5orf51, CALU, CBX5, CCNT2, CDKN1A, CDV3, CEP135, CLDN12, CRY2, DNA2, DVL3, E2F6, EDEM3, EIF4G2, EPHA4, ESPL1, FAM104A, FNDC3A, GNG5, GOLGA4, GPAT4, **HMGA1**, HMGA2, **IGF1R**, **IGF2BP1,** IL13, KMT2D, LIMD2, **LRIG3**, **MAP3K1**, MAPK6, **MARS2**, MBD2, MDM4, MLLT10, MSI2, MTUS1, MXD1, NAA30, **NAP1L1**, NAT8L, NR6A1, ONECUT2, PCGF3, PDE12, PDP2, PEG10, PEX11B, PGM2L1, PLAGL2, PLD3, PLEKHO1, PLXND1, POLR3D, POTEM, **PPP1R15B**, PPP2R2A, PRSS22, RAB11FIP4, RAB40C, RANBP2, RBFOX2, **RDX**, RNF44, RRM2, SEMA4C, SLC10A7, SLC20A1, SLC5A6, SMAD2, SMARCAD1, SMC1A, **SMCR8**, STRN, STX3, SURF4, SYT1, **TGFBR3**, TMED5, USP38, **YOD1**, ZBTB5, **ZNF644.**
**miRNA Downregulated**	**mRNA Targets**
hsa-miR-371a-5p	ACVR1B, CDC73, DCP2, GLRX2, IKZF3, MYCN, NAA50, SOX2, SYNJ2BP.
hsa-miR-365a-3p	ACVR1, BCL2, CHD8, FMC1-LUC7L2, LUC7L2, MYLIP, NUFIP2, SGK1, VGLL4, XPO4, ZNF148, **ZNF644.**
hsa-miR-205-5p	ACSL1, AMOT, B4GALT5, B4GALT6, CCNJ, CENPF, CREB1, DDX5, E2F1, ENPP4, ERBB3, ETNK1, EZR, LAMC1, LCOR, LPCAT1, LRP1, LRRK2, LYSMD3, MMD, NOTCH2, PARD6B, PHC2, PICALM, PRKCE, PTPRJ, PTPRM, RAB11FIP1, RAP2B, RTN3, RUNX2, SATB2, SMAD1, TM9SF2, VEGFA, YES1, ZEB1, ZEB2.
hsa-miR-196a-5p	BACH1, BRAP, CCDC47, CPD, CPEB3, EPHA7, EXOC8, FAM104A, GATA6, GLTP, GRPEL2, **HMGA1**, HOXA7, HOXC8, IGDCC4, **IGF2BP1**, IGF2BP3, KCTD21, LCOR, LIN28B, **LRIG3**, **MARS2**, MIEF1, **NAP1L1**, NRXN1, NXPE3, **PPP1R15B**, RCC2, **RDX**, RGL2, RTL8A, **SMCR8**, **TGFBR3**, TSPAN12, **YOD1**.
hsa-miR-141-3p	ATXN7L1, BRD3, CLOCK, E2F3, ELAVL2, EPHA7, GATA6, HNRNPF, HOXB5, **IGF1R**, IRF2BPL, KEAP1, KLF12, LPP, MARCHF6, OGT, PHLPP2, **PPP1R15B**, PRELID2, PTEN, PTPRD, QKI, RAP2C, SCD5, SLC35D1, STAT5A, TET1, TNKS2, YAP1, YWHAG, ZEB1, ZEB2, ZMPSTE24.
hsa-miR-208a-3p	ETS1, MED13.
hsa-miR-302b-3p	ADAT2, ARID4B, BTG1, CADM2, CCND2, CDK2, CREBRF, CRK, CUL3, DAZAP2, EGFR, ELK4, ESR1, FEM1C, GDF11, HDAC4, HIVEP2, HOOK3, IRF2, KLF3, KREMEN1, LCLAT1, LEFTY1, **MAP3K1**, MBNL3, MIXL1, MYO1D, NFIB, NR2F2, OSTM1, PAK2, POU2F1, PRRG4, PSD3, RAB11FIP1, RASGEF1A, RGMB, SAMD12, SBNO1, SIK1, SLAIN2, SLC22A23, SPRED1, SUCO, SUZ12, TAOK1, TNKS2, ULK1, UNK, WEE1, **YOD1,** ZBTB18, ZFYVE26, ZNF385A, ZNFX1.
hsa-miR-374a-5p	CTDSPL2, IKZF2, MBNL2, PELI1, RC3H1, SEC23B, TACC1, TFDP1.

Highlighted in bold are the genes in common. A list of abbreviations is present in Supplementary Materials.

**Table 3:** KEGG pathways. Significant KEGG pathways and genes that might be regulated by upregulated miRNA (in bold) and downregulated miRNAs (not in bold) and participate in the pathogenesis of lipedema.

ID	Pathway	Count	Genes	Fold Enrichment
**hsa04110**	**Cell cycle**	**4**	***SMC1A, CDKN1A, SMAD2, ESPL1***	**3.22**
**hsa04114**	**Oocyte meiosis**	**3**	***SMC1A, IGF1R, ESPL1***	**1.48**
**hsa05321**	**Inflammatory bowel disease**	**2**	***SMAD2, IL13***	**1.32**
hsa01522	Endocrine resistance	7	*E2F3, IGF1R, E2F1, NOTCH2, ESR1, EGFR, BCL2*	4.02
hsa01521	EGFR tyrosine kinase inhibitor resistance	3	*PTEN, IGF1R, VEGFA, ERBB3, EGFR, BCL2*	3.66
hsa04510	Focal adhesion	9	*PTEN, IGF1R, VEGFA, LAMC1, CRK, PAK2, EGFR, CCND2, BCL2*	3.46
hsa04110	Cell cycle	7	*E2F3, YWHAG, E2F1, WEE1, CCND2, CDK2, TFDP1*	3.32
hsa04350	TGF-β signaling pathway	6	*SMAD1, RGMB, LEFTY1, ACVR1, ACVR1B, TFDP1*	3.22
hsa04390	Hippo signaling pathway	7	*YWHAG, YAP1, PARD6B, SMAD1, AMOT, CCND2, SOX2*	2.78
hsa04068	FoxO signaling pathway	6	*PTEN, IGF1R, EGFR, CCND2, CDK2, SGK1*	2.50
hsa04933	AGE-RAGE signaling pathway in diabetic complications	4	*STAT5A, VEGFA, PRKCE, BCL2*	1.61
hsa04934	Cushing syndrome	4	*E2F3, CREB1, E2F1, EGFR, CDK2*	1.55
hsa04931	Insulin resistance	4	*OGT, PTEN, CREB1, PRKCE*	1.51
hsa04066	HIF-1 signaling pathway	4	*IGF1R, VEGFA, EGFR, BCL2*	1.49
hsa04917	Prolactin signaling pathway	3	*STAT5A, ESR1, CCND2*	1.38
hsa04152	AMPK signaling pathway	4	*SCD5, IGF1R, CREB1, ULK1*	1.35

## Data Availability

Data are available upon request to the corresponding authors.

## Supplementary Materials

The following supporting information can be downloaded at: https://www.mdpi.com/article/10.3390/cimb46110710/s1.
