# Supplementary material for: Identification of Specific microRNAs in Adipose Tissue Affected by Lipedema
Source: Curr Issues Mol Biol. 2024 Oct 25;46(11):11957–74. doi: 10.3390/cimb46110710 (PMC11592672; doi:10.3390/cimb46110710)
Supplement: Supplementary file 1 [file cimb-46-00710-s001.zip › cimb-3197934-supplementary.pdf]

Abbreviation

ABHD17C: Abhydrolase Domain Containing 17C

ACER2: Alkaline Ceramidase 2

ACSL1: Acyl-CoA Synthetase Long Chain Family Member 1

ACVR1: Activin A Receptor Type 1

ACVR1B: Activin A Receptor Type 1B

ADAT2: Adenosine Deaminase, tRNA-Specific 2

AGO1: Argonaute RISC Catalytic Component 1

AKR1C1: Aldo-Keto Reductase Family 1 Member C1

AMOT: Angiomotin

AP1S1: Adaptor-Related Protein Complex 1 Subunit Sigma 1

ARID3A: AT-Rich Interaction Domain 3A

ARID3B: AT-Rich Interaction Domain 3B

ARID4B: AT-Rich Interaction Domain 4B

ATXN7L1: Ataxin 7 Like 1

B4GALT5: Beta-1,4-Galactosyltransferase 5

B4GALT6: Beta-1,4-Galactosyltransferase 6

BACH1: BTB Domain and CNC Homolog 1

BCL2: BCL2 Apoptosis Regulator

BCL2L1: BCL2-Like 1 (also known as BCL-XL)

BEND4: BEN Domain-Containing Protein 4

BP: Biological Processes

BRAP: BRCA1 Associated Protein

BRD3: Bromodomain Containing 3

BTG1: B-Cell Translocation Gene 1

BZW1: Basic Leucine Zipper and W2 Domain-Containing Protein 1

C19orf47: Chromosome 19 Open Reading Frame 47

C5orf51: Chromosome 5 Open Reading Frame 51

CADM2: Cell Adhesion Molecule 2

CALU: Calumenin

CBX5: Chromobox Protein Homolog 5 (also known as HP1α)

CC: Cellular Component

CCDC47: Coiled-Coil Domain Containing 47

CCND2: Cyclin D2

CCNJ: Cyclin J

CCNT2: Cyclin T2

CDC73: Cell Division Cycle 73

CDK2: Cyclin-Dependent Kinase 2

CDKN1A: Cyclin-Dependent Kinase Inhibitor 1A (also known as p21)

CDV3: CDV3 Homolog (Cell Division Cycle 3-Like Protein)

CENPF: Centromere Protein F

CEP135: Centrosomal Protein 135

CHD8: Chromodomain Helicase DNA Binding Protein 8

CLDN12: Claudin 12

CLOCK: Circadian Locomotor Output Cycles Kaput

CPD: Carboxypeptidase D

CPEB3: Cytoplasmic Polyadenylation Element Binding Protein 3

CREB1: cAMP Response Element-Binding Protein 1

CREBRF: CREB3 Regulatory Factor

CRK: CRK Proto-Oncogene, Adaptor Protein

CRY2: Cryptochrome 2

CTDSPL2: CTD Small Phosphatase Like 2

CUL3: Cullin 3

DAZAP2: DAZ Associated Protein 2

DCP2: Decapping mRNA 2

DDX5: DEAD-Box Helicase 5

DNA2: DNA Replication Helicase/Nuclease 2

DVL3: Dishevelled Segment Polarity Protein 3

E2F1: E2F Transcription Factor 1

E2F3: E2F Transcription Factor 3

E2F6: E2F Transcription Factor 6

EDEM3: ER Degradation Enhancing Alpha-Mannosidase-Like Protein 3

EGFR: Epidermal Growth Factor Receptor

EIF4G2: Eukaryotic Translation Initiation Factor 4 Gamma 2

ELAVL2: ELAV Like RNA Binding Protein 2

ELK4: ETS Transcription Factor ELK4

ENPP4: Ectonucleotide Pyrophosphatase/Phosphodiesterase 4

EPHA4: Ephrin Type-A Receptor 4

EPHA7: Ephrin Type-A Receptor 7

ERBB: Erb-B2 Receptor

ERBB3: Erb-B2 Receptor Tyrosine Kinase 3

ESPL1: Extra Spindle Pole Bodies Like 1

ESR1: Estrogen Receptor 1

ETNK1: Ethanolamine Kinase 1

ETS1: ETS Proto-Oncogene 1

EXOC8: Exocyst Complex Component 8

EZR: Ezrin

FAM104A: Family With Sequence Similarity 104 Member A

FEM1C: Fem-1 Homolog C

FMC1-LUC7L2: FMC1-Like Cwf 1, Cell Cycle Control (S. Pombe) Domain Containing LUC7L2

FNDC3A: Fibronectin Type III Domain Containing 3A

GATA6: GATA Binding Protein 6

GDF11: Growth Differentiation Factor 11

GLRX2: Glutaredoxin 2

GLTP: Glycolipid Transfer Protein

GNG5: G Protein Subunit Gamma 5

GOLGA4: Golgin A4

GPAT4: Glycerol-3-Phosphate Acyltransferase 4

GRPEL2: GrpE-Like 2, Mitochondrial

HDAC4: Histone Deacetylase 4

HIVEP2: Human Immunodeficiency Virus Type I Enhancer Binding Protein 2

HMGA1: High Mobility Group AT-Hook 1

HMGA2: High Mobility Group AT-Hook 2

HNRNPF: Heterogeneous Nuclear Ribonucleoprotein F

HOOK3: Hook Microtubule-Tethering Protein 3

HOXA7: Homeobox A7

HOXB5: Homeobox B5

HOXC8: Homeobox C8

IGDCC4: Immunoglobulin Superfamily, DCC Subclass, Member 4

IGF1R: Insulin-Like Growth Factor 1 Receptor

IGF2BP1: Insulin-Like Growth Factor 2 mRNA-Binding Protein 1

IGF2BP3: Insulin-Like Growth Factor 2 mRNA-Binding Protein 3

IKZF2: IKAROS Family Zinc Finger 2

IKZF3: IKAROS Family Zinc Finger 3

IL13: Interleukin 13

IRF2: Interferon Regulatory Factor 2

IRF2BPL: Interferon Regulatory Factor 2 Binding Protein-Like

KCTD21: Potassium Channel Tetramerization Domain Containing 21

KEAP1: Kelch-Like ECH-Associated Protein 1

KLF12: Kruppel-Like Factor 12

KLF3: Kruppel-Like Factor 3

KMT2D: Lysine Methyltransferase 2D (also known as MLL2)

KREMEN1: Kringle Containing Transmembrane Protein 1

LAMC1: Laminin Subunit Gamma 1

LCLAT1: Lysocardiolipin Acyltransferase 1

LCOR: Ligand-Dependent Nuclear Receptor Corepressor

LEFTY1: Left-Right Determination Factor 1

LIMD2: LIM Domain Containing 2

LIN28B: Lin-28 Homolog B

LPCAT1: Lysophosphatidylcholine Acyltransferase 1

LPP: LIM Domain Containing Preferred Translocation Partner in Lipoma

LRIG3: Leucine-Rich Repeats and Immunoglobulin-Like Domains 3

LRP1: Low-Density Lipoprotein Receptor-Related Protein 1

LRRK2: Leucine-Rich Repeat Kinase 2

LUC7L2: LUC7 Like Splicing Factor 2

LYSMD3: LysM, Putative Peptidoglycan-Binding, Domain-Containing 3

MAP3K1: Mitogen-Activated Protein Kinase Kinase Kinase 1

MAPK6: Mitogen-Activated Protein Kinase 6

MARCHF6: Membrane Associated Ring-CH-Type Finger 6

MARS2: Methionyl-tRNA Synthetase 2, Mitochondrial

MBD2: Methyl-CpG Binding Domain Protein 2

MBNL2: Muscleblind Like Splicing Regulator 2

MBNL3: Muscleblind-Like Splicing Regulator 3

MDM4: MDM2 Proto-Oncogene, E3 Ubiquitin Protein Ligase Binding Protein

MED13: Mediator Complex Subunit 13

MF: Molecular Function

MIEF1: Mitochondrial Elongation Factor 1

miR: microRNA

miRNAs: microRNAs

MIXL1: Mix Paired-Like Homeobox

MLLT10: Super Elongation Complex Subunit (also known as AF10)

MMD: Monocyte to Macrophage Differentiation-Associated

MSI2: Musashi RNA Binding Protein 2

MTUS1: Microtubule-Associated Tumor Suppressor 1

MXD1: MAX Dimerization Protein 1

MYCN: MYCN Proto-Oncogene, BHLH Transcription Factor

MYLIP: Myosin Regulatory Light Chain Interacting Protein

MYO1D: Myosin ID

NAA30: N(alpha)-Acetyltransferase 30, NatC Catalytic Subunit

NAA50: N-alpha-Acetyltransferase 50

NAP1L1: Nucleosome Assembly Protein 1-Like 1

NAT8L: N-Acetyltransferase 8-Like

NFIB: Nuclear Factor I/B

NOTCH2: Notch Receptor 2

NR2F2: Nuclear Receptor Subfamily 2 Group F Member 2

NR6A1: Nuclear Receptor Subfamily 6 Group A Member 1

NRXN1: Neurexin 1

NUFIP2: Nuclear FMRP Interacting Protein 2

NXPE3: Neurexophilin and PC-esterase Domain Family Member 3

OGT: O-Linked N-Acetylglucosamine Transferase

ONECUT2: One Cut Homeobox 2

OSTM1: Osteoclastogenesis-Associated Transmembrane Protein 1

PAK2: p21-Activated Kinase 2

PARD6B: Par-6 Family Cell Polarity Regulator Beta

PCGF3: Polycomb Group RING Finger 3

PDE12: Phosphodiesterase 12

PDP2: Pyruvate Dehyrogenase Phosphatase Catalytic Subunit 2

PEG10: Paternally Expressed 10

PELI1: Pellino E3 Ubiquitin Protein Ligase 1

PEX11B: Peroxisomal Biogenesis Factor 11 Beta

PF4: Platelet Factor 4

PGM2L1: Phosphoglucomutase 2-Like 1

PHC2: Polyhomeotic Homolog 2

PHLPP2: PH Domain and Leucine Rich Repeat Protein Phosphatase 2

PICALM: Phosphatidylinositol Binding Clathrin Assembly Protein

PLAGL2: PLAG1 Like Zinc Finger 2

PLD3: Phospholipase D Family Member 3

PLEKHO1: Pleckstrin Homology Domain Containing O1

PLXND1: Plexin D1

POLR3D: RNA Polymerase III Subunit D

POTEM: POTE Ankyrin Domain Family Member M

POU2F1: POU Class 2 Homeobox 1

PPP1R15B: Protein Phosphatase 1 Regulatory Subunit 15B

PPP2R2A: Protein Phosphatase 2 Regulatory Subunit B Alpha

PRELID2: PRELI Domain Containing 2

PRKCE: Protein Kinase C Epsilon

PRRG4: Proline Rich and Gla Domain 4

PRSS22: Protease, Serine 22

PSD3: Pleckstrin and Sec7 Domain Containing 3

PTEN: Phosphatase and Tensin Homolog

PTPRD: Protein Tyrosine Phosphatase, Receptor Type D

PTPRJ: Protein Tyrosine Phosphatase, Receptor Type J

PTPRM: Protein Tyrosine Phosphatase, Receptor Type M

QKI: KH Domain Containing RNA Binding

RAB11FIP1: RAB11 Family Interacting Protein 1

RAB11FIP4: RAB11 Family Interacting Protein 4

RAB40C: RAB40C, Member RAS Oncogene Family

RANBP2: RAN Binding Protein 2

RAP2B: Member of RAS Oncogene Family

RAP2C: Member of RAS Oncogene Family

RASGEF1A: RasGEF Domain Family Member 1A

RBFOX2: RNA Binding Fox-1 Homolog 2

RC3H1: Ring Finger and CCCH-Type Domains 1

RCC2: Regulator of Chromosome Condensation 2

RDX: Radixin

RGL2: Ral Guanine Nucleotide Dissociation Stimulator-Like 2

RGMB: Repulsive Guidance Molecule BMP Co-Receptor

RNF44: Ring Finger Protein 44

RRM2: Ribonucleotide Reductase Regulatory Subunit M2

RTL8A: Retrotransposon Gag Like 8A

RTN3: Reticulon 3

RUNX2: Runt-Related Transcription Factor 2

SAMD12: Sterile Alpha Motif Domain Containing 12

SATB2: Special AT-Rich Sequence-Binding Protein 2

SBNO1: Strawberry Notch Homolog 1

SCD5: Stearoyl-CoA Desaturase 5

SEC23B: Sec23 Homolog B, COPII Coat Complex Component

SEMA4C: Semaphorin 4C

SGK1: Serum/Glucocorticoid Regulated Kinase 1

SIK1: Salt-Inducible Kinase 1

SLAIN2: SLAIN Motif Family Member 2

SLC10A7: Solute Carrier Family 10 Member 7

SLC20A1: Solute Carrier Family 20 Member 1

SLC22A23: Solute Carrier Family 22 Member 23

SLC35D1: Solute Carrier Family 35 Member D1

SLC5A6: Solute Carrier Family 5 Member 6

SMAD1: SMAD Family Member 1

SMAD2: SMAD Family Member 2

SMARCAD1: SWI/SNF-Related, Matrix-Associated Actin-Dependent Regulator of Chromatin, Subfamily A, Containing DEAD/H Box 1

SMC1A: Structural Maintenance of Chromosomes 1A

SMCR8: Smith-Magenis Syndrome Chromosome Region, Candidate 8

SOX2: SRY-Box Transcription Factor 2

SPRED1: Sprouty-Related EVH1 Domain-Containing Protein 1

STAT5A: Signal Transducer and Activator of Transcription 5A

STRN: Striatin

STX3: Syntaxin 3

SUCO: SUN Domain Containing Ossification Factor

SURF4: Surfeit 4

SUZ12: SUZ12 Polycomb Repressive Complex 2 Subunit

SYNJ2BP: Synaptojanin 2 Binding Protein

SYT1: Synaptotagmin 1

TACC1: Transforming Acidic Coiled-Coil Containing Protein 1

TAOK1: TAO Kinase 1

TET1: Tet Methylcytosine Dioxygenase 1

TFDP1: Transcription Factor Dp-1

TGFBR3: Transforming Growth Factor Beta Receptor 3

TM9SF2: Transmembrane 9 Superfamily Member 2

TMED5: Transmembrane P24 Trafficking Protein 5

TNKS2: Tankyrase 2

TSPAN12: Tetraspanin 12

ULK1: Unc-51 Like Autophagy Activating Kinase 1

UNK: UNK Proto-Oncogene

USP38: Ubiquitin Specific Peptidase 38

VEGFA: Vascular Endothelial Growth Factor A

VGLL4: Vestigial Like Family Member 4

WEE1: WEE1 G2 Checkpoint Kinase

XPO4: Exportin 4

YAP1: Yes-Associated Protein 1

YES1: YES Proto-Oncogene 1

YOD1: Deubiquitinase OTU1

YWHAG: 14-3-3 Protein Gamma

ZBTB18: Zinc Finger and BTB Domain Containing 18

ZBTB5: Zinc Finger and BTB Domain Containing 5

ZEB1: Zinc Finger E-Box Binding Homeobox 1

ZEB2: Zinc Finger E-Box Binding Homeobox 2

ZFYVE26: Zinc Finger FYVE-Type Containing 26

ZMPSTE24: Zinc Metallopeptidase STE24

ZNF148: Zinc Finger Protein 148

ZNF385A: Zinc Finger Protein 385A

ZNF644: Zinc Finger Protein 644

ZNFX1: Zinc Finger NFX1-Type Containing 1

Table S1. List of validated target genes upregulated miRNAs. Based on miRwalk database. miRTarbase as experimental validated platform. TargetScan and miRDB as predicted target platform.

Gene symbol	miRTarbase	TargetScan	miRDB
C19orf47	MIRT559083	1	1
RDX	MIRT521388	1	1
YOD1	MIRT510517	1	1
E2F6	MIRT567713	1	1
MDM4	MIRT112245	1	1
NR6A1	MIRT471964	1	1
SURF4	MIRT565447	1	1
SMC1A	MIRT505578	1	1
CDV3	MIRT255318	1	1
AP1S1	MIRT481850	1	1
FAM104A	MIRT574784	1	1
USP38	MIRT553015	1	1
PLD3	MIRT735748	1	1
IGF1R	MIRT073672	1	1
SYT1	MIRT735754	1	1
RAB11FIP4	MIRT501436	1	1
EPHA4	MIRT548349	1	1
ARID3B	MIRT073008	1	1
AGO1	MIRT046562	1	1
SLC10A7	MIRT501177	1	1
BCL2L1	MIRT004490	1	1
EDEM3	MIRT704033	1	1
LIMD2	MIRT493327	1	1

ZNF644	MIRT505021	1	1
PPP1R15B	MIRT470420	1	1
CCNT2	MIRT548973	1	1
CDKN1A	MIRT218816	1	1
MARS2	MIRT735743	1	1
MSI2	MIRT547376	1	1
NAP1L1	MIRT712368	1	1
SMCR8	MIRT076227	1	1
HMGA1	MIRT168587	1	1
LRIG3	MIRT547574	1	1
PGM2L1	MIRT471273	1	1
C5orf51	MIRT215724	1	1
NAT8L	MIRT309849	1	1
GPAT4	MIRT735740	1	1
BEND4	MIRT549210	1	1
MAPK6	MIRT193104	1	1
STRN	MIRT500905	1	1
SLC5A6	MIRT501114	1	1
KMT2D	MIRT502131	1	1
PPP2R2A	MIRT322431	1	1
SEMA4C	MIRT501244	1	1
CALU	MIRT123321	1	1
STX3	MIRT761048	1	1
SMAD2	MIRT054664	1	1
RBFOX2	MIRT501391	1	1
MLLT10	MIRT473130	1	1
GOLGA4	MIRT574745	1	1
ZBTB5	MIRT491858	1	1
RNF44	MIRT501353	1	1
PLXND1	MIRT470863	1	1

MBD2	MIRT501947	1	1
PLEKHO1	MIRT498120	1	1
SMARCAD1	MIRT094464	1	1
MTUS1	MIRT472734	1	1
PDP2	MIRT284531	1	1
CRY2	MIRT180606	1	1
RAB40C	MIRT469716	1	1
ABHD17C	MIRT568659	1	1
PRSS22	MIRT700591	1	1
CEP135	MIRT502786	1	1
IL13	MIRT006399	1	1
MXD1	MIRT303323	1	1
PLAGL2	MIRT485294	1	1
TGFBR3	MIRT546331	1	1
HMGA2	MIRT002097	1	1
PEX11B	MIRT266216	1	1
DVL3	MIRT523988	1	1
ONECUT2	MIRT080402	1	1
ARID3A	MIRT494683	1	1
GNG5	MIRT567433	1	1
SLC20A1	MIRT297445	1	1
MAP3K1	MIRT046560	1	1
RANBP2	MIRT046567	1	1
PCGF3	MIRT094164	1	1
IGF2BP1	MIRT003831	1	1
CBX5	MIRT549023	1	1
CLDN12	MIRT260711	1	1
ESPL1	MIRT065691	1	1
PDE12	MIRT120924	1	1
EIF4G2	MIRT024118	1	1

POLR3D	MIRT123980	1	1
ACER2	MIRT324735	1	1
NAA30	MIRT191407	1	1
RRM2	MIRT046565	1	1
PEG10	MIRT561780	1	1
FNDC3A	MIRT068792	1	1
DNA2	MIRT524114	1	1
POTEM	MIRT735751	1	1
TMED5	MIRT466187	1	1
BZW1	MIRT300041	1	1

Table S2. List of validated target genes dowregulated miRNAs. Based on miRwalk database. miRTarbase as experimental validated platform. TargetScan and miRDB as predicted target platform.

Gene symbol	miRTarbase	TargetScan	miRDB
PHLPP2	MIRT054812	1	1
QKI	MIRT099339	1	1
PTPRD	MIRT021661	1	1
ATXN7L1	MIRT539040	1	1
OGT	MIRT176070	1	1
E2F3	MIRT021649	1	1
ELAVL2	MIRT537734	1	1
KLF12	MIRT731956	1	1
KEAP1	MIRT053761	1	1
YWHAG	MIRT006730	1	1
EPHA7	MIRT548307	1	1
BRD3	MIRT003279	1	1

YAP1	MIRT052906	1	1
TET1	MIRT382486	1	1
RAP2C	MIRT762642	1	1
IRF2BPL	MIRT737588	1	1
HOXB5	MIRT021658	1	1
CLOCK	MIRT002951	1	1
PTEN	MIRT003281	1	1
SCD5	MIRT718597	1	1
IGF1R	MIRT733327	1	1
PRELID2	MIRT498346	1	1
GATA6	MIRT548122	1	1
STAT5A	MIRT731071	1	1
ZEB1	MIRT000317	1	1
MARCHF6	MIRT506629	1	1
ZMPSTE24	MIRT438256	1	1
HNRNPF	MIRT502258	1	1
TNKS2	MIRT127534	1	1
LPP	MIRT702208	1	1
PPP1R15B	MIRT061757	1	1
ZEB2	MIRT000316	1	1
SLC35D1	MIRT554195	1	1
RDX	MIRT054025	1	1
FAM104A	MIRT026086	1	1
CPD	MIRT026089	1	1
MIEF1	MIRT026065	1	1
MARS2	MIRT470426	1	1
NRXN1	MIRT738653	1	1
NAP1L1	MIRT048156	1	1
SMCR8	MIRT522366	1	1
HMGA1	MIRT076226	1	1

GRPEL2	MIRT006802	1	1
LRIG3	MIRT095769	1	1
EXOC8	MIRT547581	1	1
BACH1	MIRT562352	1	1
BRAP	MIRT004718	1	1
CCDC47	MIRT559183	1	1
TGFBR3	MIRT026099	1	1
IGDCC4	MIRT546336	1	1
LIN28B	MIRT026071	1	1
CPEB3	MIRT026096	1	1
GLTP	MIRT026085	1	1
YOD1	MIRT048270	1	1
RCC2	MIRT520041	1	1
HOXC8	MIRT546787	1	1
RGL2	MIRT002942	1	1
IGF2BP1	MIRT026092	1	1
IGF2BP3	MIRT492430	1	1
HOXA7	MIRT502410	1	1
TSPAN12	MIRT026098	1	1
LCOR	MIRT026059	1	1
KCTD21	MIRT002940	1	1
RTL8A	MIRT026095	1	1
NXPE3	MIRT057645	1	1
ETNK1	MIRT474917	1	1
LPCAT1	MIRT493925	1	1
PTPRJ	MIRT547253	1	1
VEGFA	MIRT133673	1	1
DDX5	MIRT440320	1	1
B4GALT6	MIRT130344	1	1
B4GALT5	MIRT004518	1	1

TM9SF2	MIRT003236	1	1
EZR	MIRT441130	1	1
CCNJ	MIRT441131	1	1
LYSMD3	MIRT712125	1	1
PARD6B	MIRT474226	1	1
CREB1	MIRT440697	1	1
SATB2	MIRT735404	1	1
YES1	MIRT003666	1	1
RAB11FIP1	MIRT440298	1	1
RAP2B	MIRT440042	1	1
PRKCE	MIRT668907	1	1
MMD	MIRT735300	1	1
PICALM	MIRT024970	1	1
RUNX2	MIRT505979	1	1
LRRK2	MIRT439820	1	1
LAMC1	MIRT003667	1	1
E2F1	MIRT440225	1	1
SMAD1	MIRT439978	1	1
PHC2	MIRT731506	1	1
LRP1	MIRT007191	1	1
PTPRM	MIRT007075	1	1
ACSL1	MIRT003322	1	1
CENPF	MIRT053377	1	1
ERBB3	MIRT439989	1	1
AMOT	MIRT001163	1	1
RTN3	MIRT002478	1	1
ENPP4	MIRT054104	1	1
NOTCH2	MIRT054854	1	1
MED13	MIRT734380	1	1
ETS1	MIRT003659	1	1

SUCO	MIRT549393	1	1
ADAT2	MIRT439720	1	1
ARID4B	MIRT440724	1	1
HOOK3	MIRT440100	1	1
MBNL3	MIRT003912	1	1
CREBRF	MIRT005541	1	1
ZNF385A	MIRT500887	1	1
ZFYVE26	MIRT539442	1	1
CRK	MIRT485832	1	1
FEM1C	MIRT244594	1	1
SLAIN2	MIRT547487	1	1
ZNFX1	MIRT165899	1	1
PAK2	MIRT532940	1	1
SAMD12	MIRT484926	1	1
KREMEN1	MIRT567938	1	1
DAZAP2	MIRT505160	1	1
WEE1	MIRT356935	1	1
SPRED1	MIRT093946	1	1
TAOK1	MIRT484836	1	1
PRRG4	MIRT505991	1	1
GDF11	MIRT243430	1	1
HDAC4	MIRT554489	1	1
OSTM1	MIRT536418	1	1
ESR1	MIRT187255	1	1
EGFR	MIRT061361	1	1
BTG1	MIRT280995	1	1
CCND2	MIRT520820	1	1
MIXL1	MIRT566284	1	1
CADM2	MIRT065865	1	1
PSD3	MIRT054097	1	1

POU2F1	MIRT640028	1	1
MYO1D	MIRT617875	1	1
ELK4	MIRT054661	1	1
HIVEP2	MIRT538862	1	1
NR2F2	MIRT003555	1	1
ZBTB18	MIRT516553	1	1
SLC22A23	MIRT450929	1	1
CDK2	MIRT571772	1	1
SIK1	MIRT383602	1	1
LCLAT1	MIRT485340	1	1
CUL3	MIRT507431	1	1
SUZ12	MIRT536874	1	1
KLF3	MIRT574566	1	1
SBNO1	MIRT561330	1	1
IRF2	MIRT467908	1	1
ULK1	MIRT733288	1	1
RGMB	MIRT057389	1	1
UNK	MIRT155900	1	1
NFIB	MIRT547633	1	1
RASGEF1A	MIRT538261	1	1
LEFTY1	MIRT242506	1	1
MAP3K1	MIRT093807	1	1
LUC7L2	MIRT521217	1	1
VGLL4	MIRT536611	1	1
SGK1	MIRT063060	1	1
ZNF644	MIRT122369	1	1
BCL2	MIRT078817	1	1
NUFIP2	MIRT513807	1	1
CHD8	MIRT534775	1	1
ZNF148	MIRT536356	1	1

XPO4	MIRT731314	1	1
MYLIP	MIRT319744	1	1
ACVR1	MIRT464227	1	1
FMC1-LUC7L2	MIRT468210	1	1
IKZF3	MIRT564633	1	1
MYCN	MIRT006243	1	1
NAA50	MIRT044042	1	1
ACVR1B	MIRT442790	1	1
SOX2	MIRT463201	1	1
DCP2	MIRT500541	1	1
CDC73	MIRT099119	1	1
SYNJ2BP	MIRT054061	1	1
GLRX2	MIRT544527	1	1
RC3H1	MIRT542081	1	1
SEC23B	MIRT535761	1	1
MBNL2	MIRT522371	1	1
CTDSPL2	MIRT065657	1	1
IKZF2	MIRT733145	1	1
PELI1	MIRT615046	1	1
TACC1	MIRT704832	1	1
TFDP1	MIRT767184	1	1

KEGG pathways regulated by the **upregulated miRNA** and participate in the pathogenesis of lipedema, in red the gene affected.

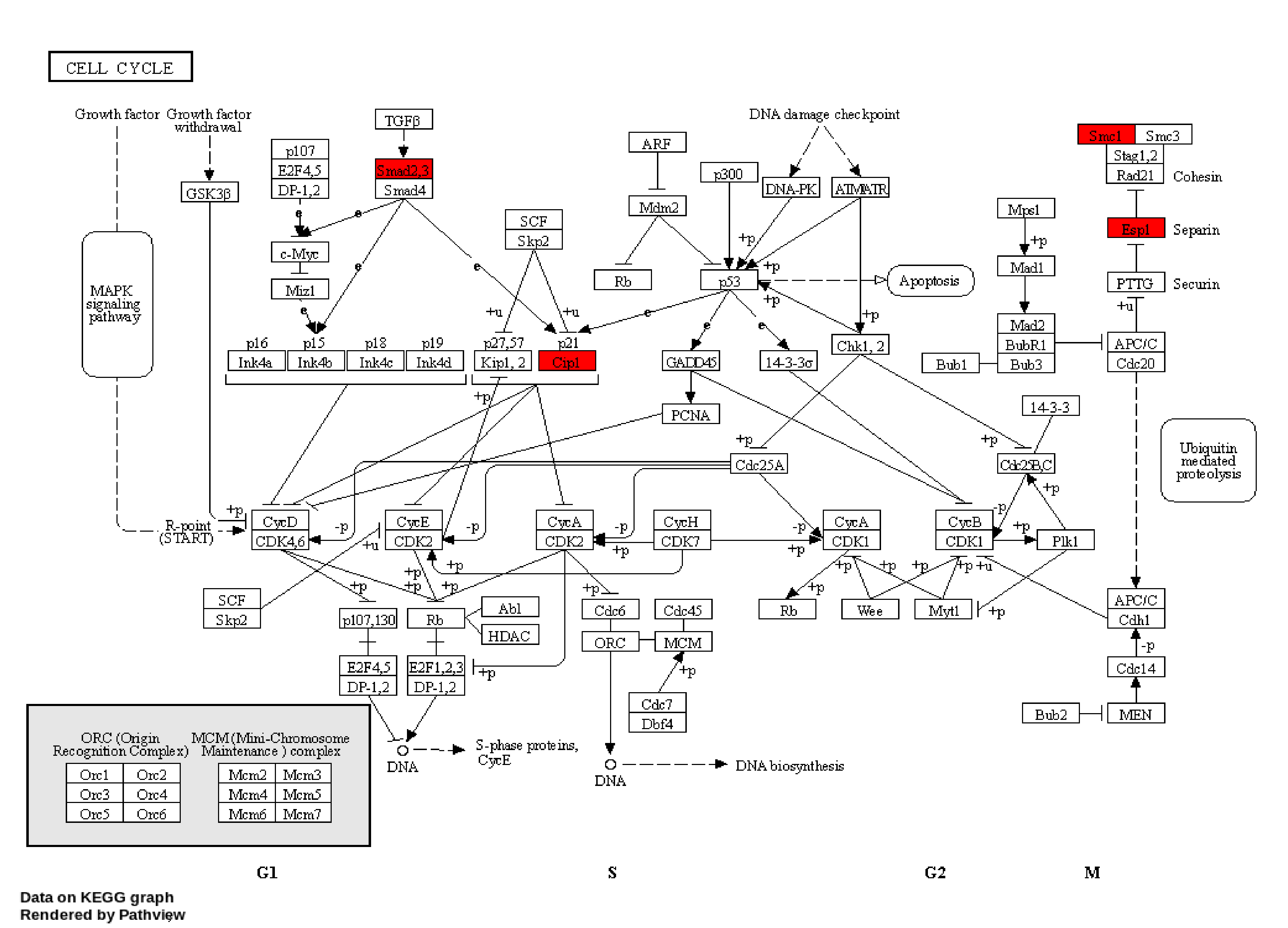

CELL CYCLE
Growth factor
Growth factor withdrawal
TGFβ
p107
E2F4,5
DP-1,2
Smad2,3
Smad4
GSK3β
c-Myc
Miz1
MAPK signaling pathway
SCF
Skp2
ARF
Mdm2
Rb
p300
DNA damage checkpoint
DNA-PK
ATM/ATR
p53
Apoptosis
Mps1
Mad1
Mad2
BubR1
Bub3
Bub1
Smc1
Smc3
Stag1,2
Rad21
Cohesin
Esp1
Separin
PTTG
Securin
APC/C
Cdc20
p16
Ink4a
p15
Ink4b
p18
Ink4c
p19
Ink4d
p27,57
Kip1, 2
p21
Cip1
GADD45
14-3-3σ
Chk1, 2
PCNA
14-3-3
Cdc25A
Cdc25B,C
Ubiquitin mediated proteolysis
R-point (START)
CycD
CDK4,6
CycE
CDK2
CycA
CDK2
CycH
CDK7
CycA
CDK1
CycB
CDK1
Plk1
SCF
Skp2
p107,130
Rb
Abl
HDAC
Cdc6
Cdc45
ORC
MCM
Rb
Wee
Myt1
APC/C
Cdh1
E2F4,5
DP-1,2
E2F1,2,3
DP-1,2
Cdc7
Dbf4
Cdc14
Bub2
MEN
DNA
S-phase proteins, CycE
DNA
DNA biosynthesis
ORC (Origin Recognition Complex)
MCM (Mini-Chromosome Maintenance) complex
Orc1 Orc2
Orc3 Orc4
Orc5 Orc6
Mcm2 Mcm3
Mcm4 Mcm5
Mcm6 Mcm7
G1
S
G2
M
Data on KEGG graph
Rendered by Pathview

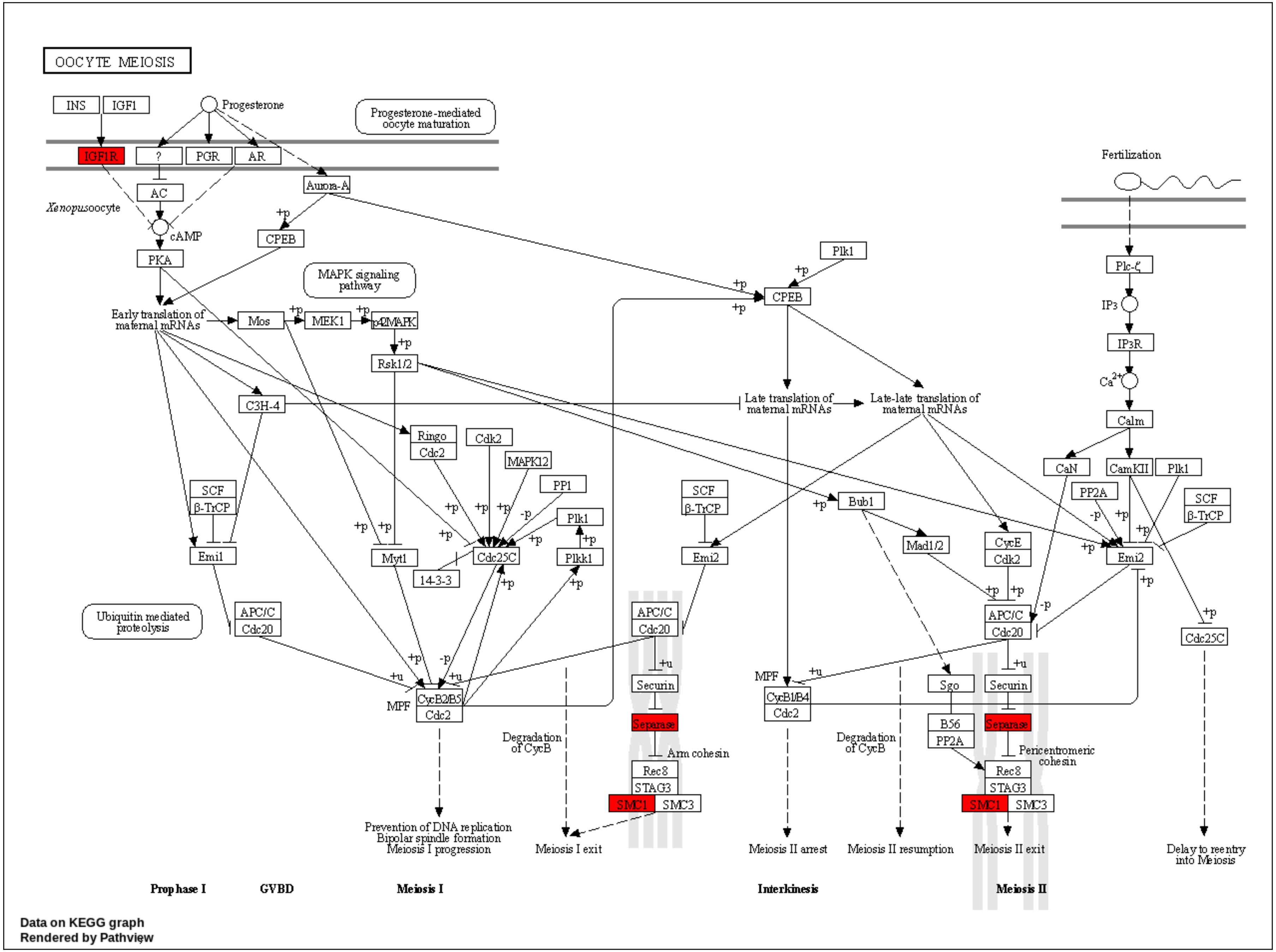
OOCYTE MEIOSIS
INS
IGF1
Progesterone
Progesterone-mediated oocyte maturation
IGF1R
?
PGR
AR
AC
Aurora-A
Xenopus oocyte
cAMP
CPEB
PKA
MAPK signaling pathway
Early translation of maternal mRNAs
Mos
MEK1
p42MAPK
Rsk1/2
C3H-4
Ringo
Cdc2
Cdk2
MAPK12
PP1
Plk1
SCF
β-TrCP
Emi1
Myt1
Cdc25C
Plkk1
14-3-3
APC/C
Cdc20
Ubiquitin mediated proteolysis
MPF
CycB2/B5
Prevention of DNA replication
Bipolar spindle formation
Meiosis I progression
Degradation of CycB
Meiosis I exit
Emi2
Securin
Separase
Arm cohesin
Rec8
STAG3
SMC1
SMC3
Late translation of maternal mRNAs
Late-late translation of maternal mRNAs
Bub1
Mad1/2
CycE
Cdk2
CycB1/B4
Meiosis II arrest
Meiosis II resumption
Sgo
B56
PP2A
Pericentromeric cohesin
Meiosis II exit
Fertilization
Plc-ζ
IP3
IP3R
Ca2+
Calm
CaN
CamKII
Delay to reentry into Meiosis
Prophase I
GVBD
Meiosis I
Interkinesis
Meiosis II
Data on KEGG graph
Rendered by Pathview

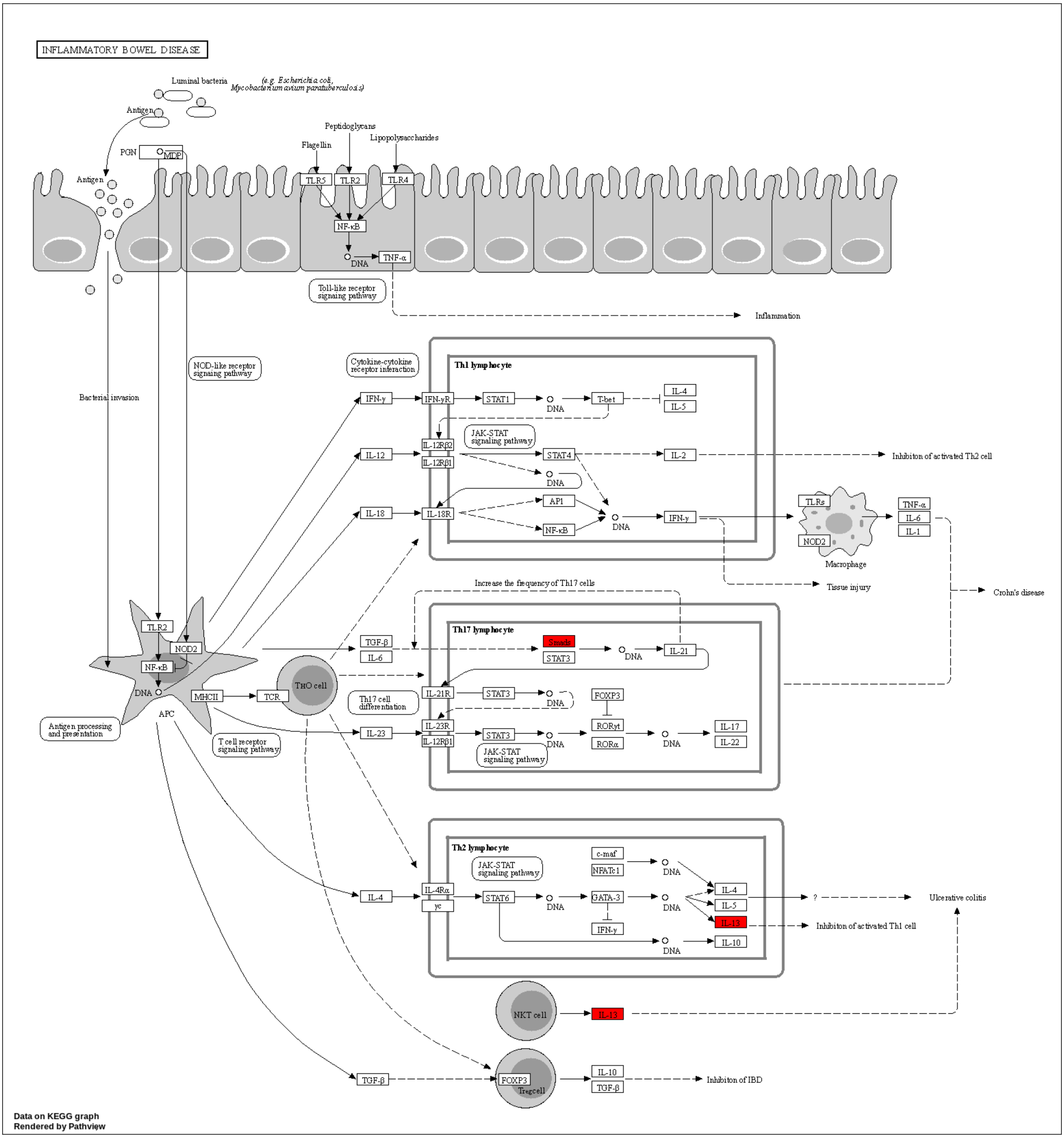
INFLAMMATORY BOWEL DISEASE
Luminal bacteria
(e.g. Escherichia coli, Mycobacterium avium paratuberculosis)
Antigen
PGN
MDP
Peptidoglycans
Flagellin
Lipopolysaccharides
TLR5
TLR2
TLR4
NF-κB
DNA
TNF-α
Toll-like receptor signaling pathway
Inflammation
NOD-like receptor signaling pathway
Bacterial invasion
Cytokine-cytokine receptor interaction
Th1 lymphocyte
IFN-γ
IFN-γR
STAT1
T-bet
IL-4
IL-5
JAK-STAT signaling pathway
IL-12
IL-12Rβ2
IL-12Rβ1
STAT4
IL-2
Inhibiton of activated Th2 cell
IL-18
IL-18R
AP1
TLRs
NOD2
Macrophage
IL-6
IL-1
Tissue injury
Crohn's disease
Increase the frequency of Th17 cells
Th17 lymphocyte
TGF-β
Smads
STAT3
IL-21
TLR2
APC
MHCII
TCR
THO cell
IL-21R
Th17 cell differentiation
FOXP3
IL-23
IL-23R
RORγt
RORα
IL-17
IL-22
Antigen processing and presentation
T cell receptor signaling pathway
Th2 lymphocyte
c-maf
NFATc1
IL-4Rα
γc
STAT6
GATA-3
IL-13
IL-10
?
Ulcerative colitis
Inhibiton of activated Th1 cell
NKT cell
Tregcell
Inhibiton of IBD
Data on KEGG graph
Rendered by Pathview

KEGG pathways regulated by the **downregulated miRNAs** and participate in the pathogenesis of lipedema, in red the gene affected.

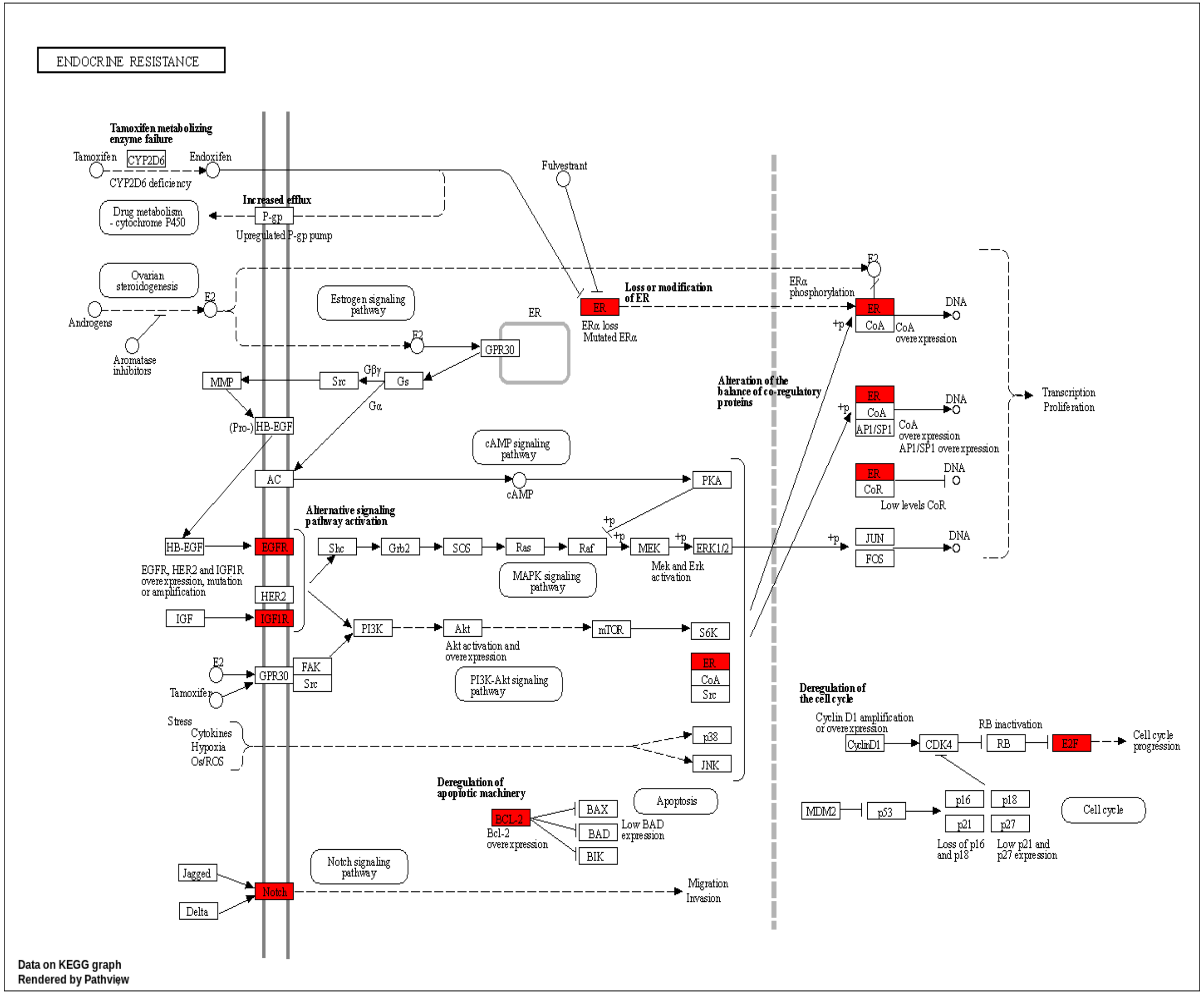
ENDOCRINE RESISTANCE
Tamoxifen metabolizing enzyme failure
Tamoxifen
CYP2D6
Endoxifen
CYP2D6 deficiency
Drug metabolism - cytochrome P450
Increased efflux
P-gp
Upregulated P-gp pump
Fulvestrant
Ovarian steroidogenesis
E2
Androgens
Aromatase inhibitors
Estrogen signaling pathway
ER
GPR30
Loss or modification of ER
ERα loss
Mutated ERα
ERα phosphorylation
CoA
CoA overexpression
DNA
MMP
Src
Gβγ
Gs
Gα
(Pro-)HB-EGF
cAMP signaling pathway
AC
cAMP
PKA
Alteration of the balance of co-regulatory proteins
AP1/SP1
CoA overexpression
AP1/SP1 overexpression
CoR
Low levels CoR
Transcription
Proliferation
Alternative signaling pathway activation
HB-EGF
EGFR
Shc
Grb2
SOS
Ras
Raf
MEK
ERK1/2
+p
JUN
FOS
MAPK signaling pathway
Mek and Erk activation
EGFR, HER2 and IGF1R overexpression, mutation or amplification
HER2
IGF
IGF1R
PI3K
Akt
mTOR
S6K
Akt activation and overexpression
PI3K-Akt signaling pathway
FAK
Src
Tamoxifen
Stress
Cytokines
Hypoxia
Os/ROS
p38
JNK
Deregulation of the cell cycle
Cyclin D1 amplification or overexpression
CyclinD1
CDK4
RB inactivation
RB
E2F
Cell cycle progression
MDM2
p53
p16
p18
p21
p27
Loss of p16 and p18
Low p21 and p27 expression
Cell cycle
Deregulation of apoptotic machinery
BCL-2
Bcl-2 overexpression
BAX
BAD
BIK
Low BAD expression
Apoptosis
Notch signaling pathway
Jagged
Notch
Delta
Migration
Invasion
Data on KEGG graph
Rendered by Pathview

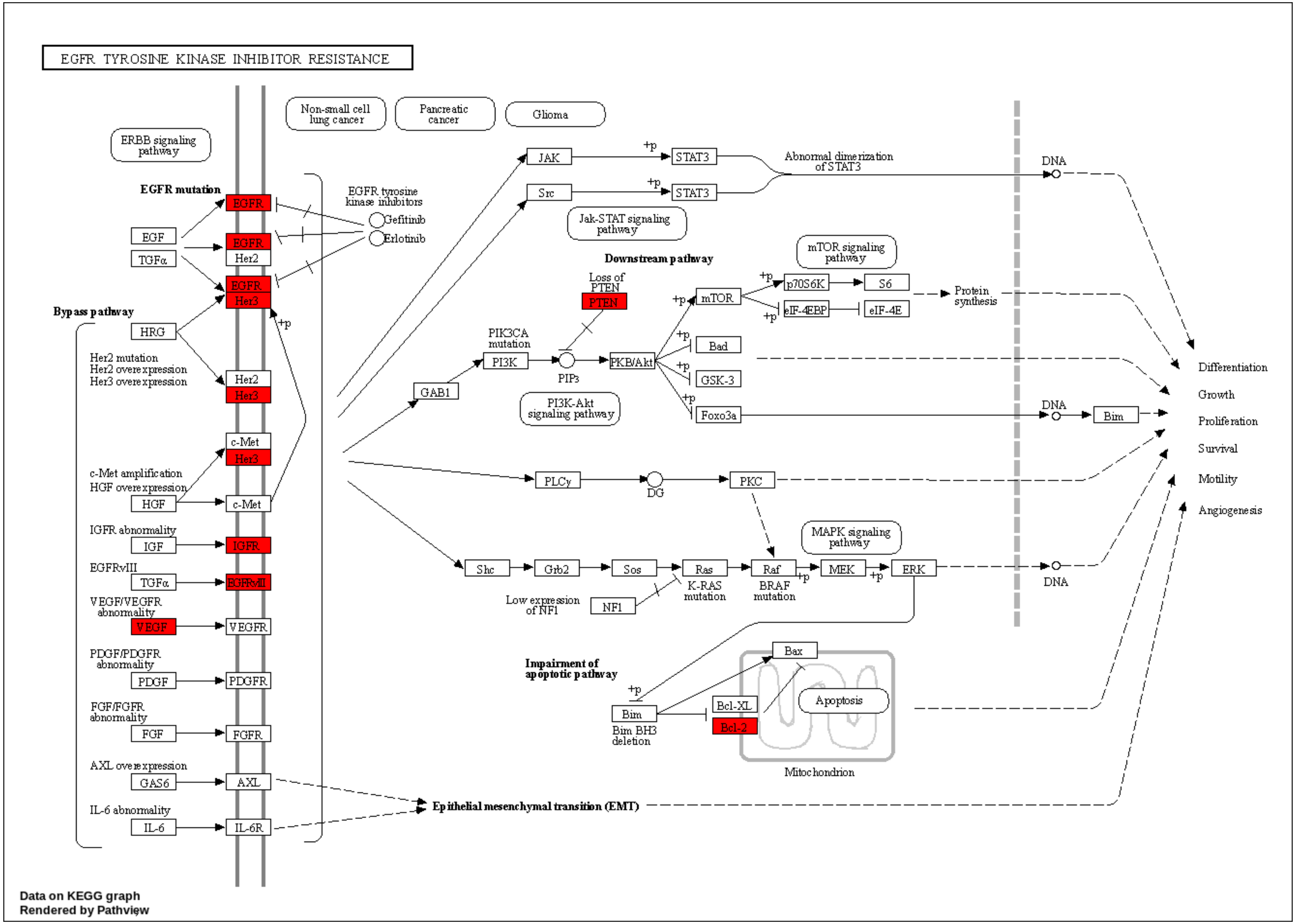
EGFR TYROSINE KINASE INHIBITOR RESISTANCE
Non-small cell lung cancer
Pancreatic cancer
Glioma
ERBB signaling pathway
EGFR mutation
EGFR
EGF
TGFα
EGFR
Her2
EGFR
Her3
EGFR tyrosine kinase inhibitors
Gefitinib
Erlotinib
Bypass pathway
HRG
Her2 mutation
Her2 overexpression
Her3 overexpression
Her2
Her3
c-Met
Her3
c-Met amplification
HGF overexpression
HGF
c-Met
IGFR abnormality
IGF
IGFR
EGFRvIII
TGFα
EGFRvIII
VEGF/VEGFR abnormality
VEGF
VEGFR
PDGF/PDGFR abnormality
PDGF
PDGFR
FGF/FGFR abnormality
FGF
FGFR
AXL overexpression
GAS6
AXL
IL-6 abnormality
IL-6
IL-6R
Epithelial mesenchymal transition (EMT)
JAK
STAT3
Src
STAT3
Abnormal dimerization of STAT3
Jak-STAT signaling pathway
Downstream pathway
mTOR signaling pathway
Loss of PTEN
PTEN
mTOR
p70S6K
S6
eIF-4EBP
eIF-4E
Protein synthesis
PIK3CA mutation
PI3K
PIP3
PKB/Akt
Bad
GSK-3
Foxo3a
GAB1
PI3K-Akt signaling pathway
PLCγ
DG
PKC
MAPK signaling pathway
Shc
Grb2
Sos
Ras
Raf
MEK
ERK
K-RAS mutation
BRAF mutation
Low expression of NF1
NF1
Impairment of apoptotic pathway
Bax
Bim
Bcl-XL
Bcl-2
Apoptosis
Bim BH3 deletion
Mitochondrion
DNA
DNA
Bim
DNA
Differentiation
Growth
Proliferation
Survival
Motility
Angiogenesis
Data on KEGG graph
Rendered by Pathview

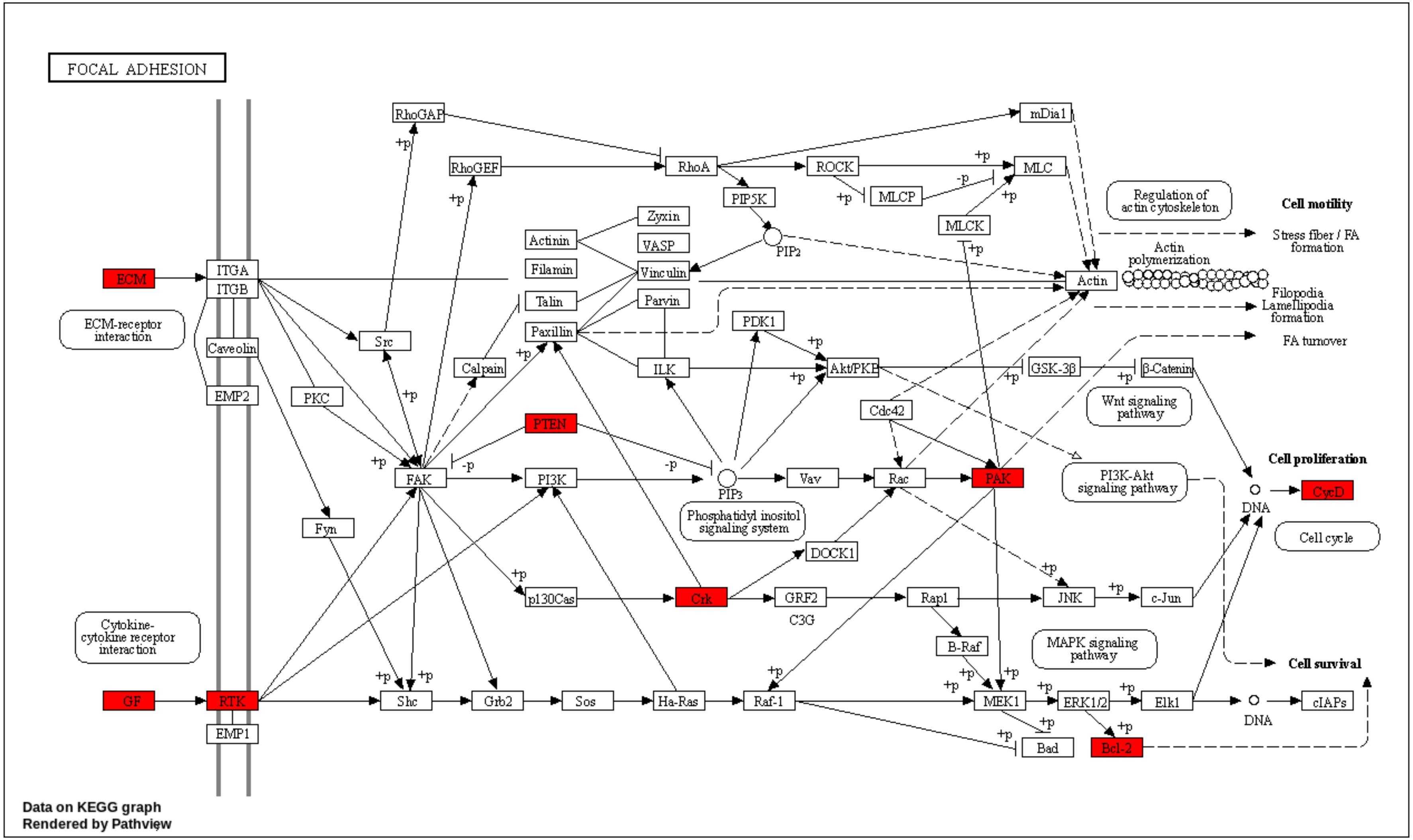
FOCAL ADHESION
ECM
ITGA
ITGB
ECM-receptor interaction
Caveolin
EMP2
PKC
Src
Fyn
RhoGAP
RhoGEF
Calpain
Actinin
Filamin
Talin
Paxillin
Zyxin
VASP
Vinculin
Parvin
ILK
PTEN
FAK
PI3K
PIP3
PIP2
PIP5K
RhoA
ROCK
MLCP
MLC
MLCK
mDia1
Regulation of actin cytoskeleton
Actin
Actin polymerization
Cell motility
Stress fiber / FA formation
Filopodia Lamellipodia formation
FA turnover
PDK1
Akt/PKB
GSK-3β
β-Catenin
Wnt signaling pathway
Cdc42
Vav
Rac
PAK
PI3K-Akt signaling pathway
Phosphatidyl inositol signaling system
DOCK1
p130Cas
Crk
GRF2
C3G
Rap1
B-Raf
JNK
c-Jun
MAPK signaling pathway
Cell proliferation
DNA
CycD
Cell cycle
Cytokine-cytokine receptor interaction
GF
RTK
EMP1
Shc
Grb2
Sos
Ha-Ras
Raf-1
MEK1
ERK1/2
Elk1
cIAPs
Bad
Bcl-2
Cell survival
Data on KEGG graph
Rendered by Pathview

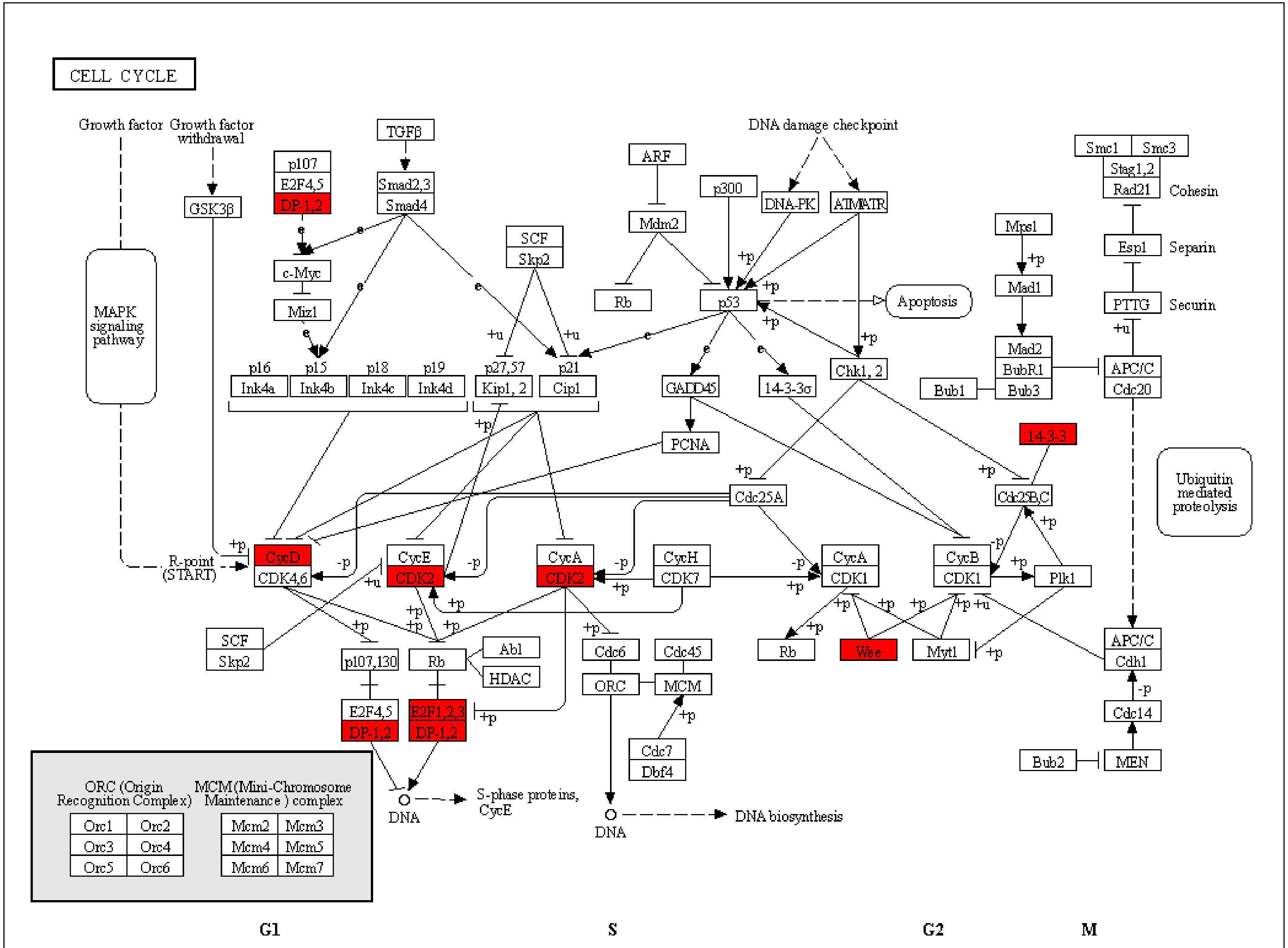

Data on KEGG graph
Rendered by Pathview

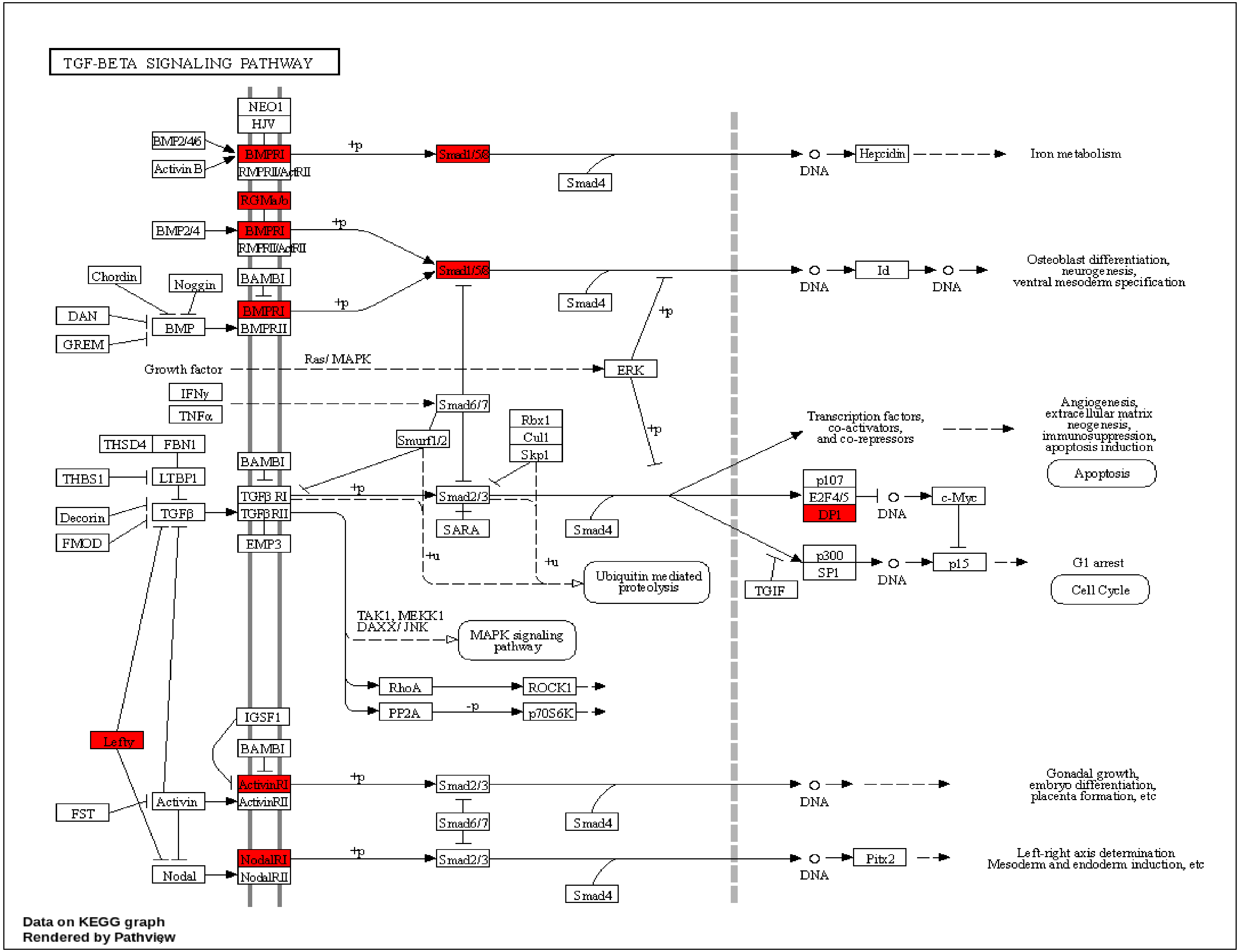
TGF-BETA SIGNALING PATHWAY
NEO1
HJV
BMP2/4/6
Activin B
BMPRI
RMPRII/ActRII
+p
Smad1/5/8
Smad4
DNA
Hepcidin
Iron metabolism
RGMa/b
BMP2/4
BMPRI
RMPRII/ActRII
+p
Smad1/5/8
Smad4
DNA
Id
DNA
Osteoblast differentiation, neurogenesis, ventral mesoderm specification
Chordin
Noggin
BAMBI
DAN
GREM
BMP
BMPRI
BMPRII
+p
Growth factor
Ras/ MAPK
ERK
+p
+p
IFNγ
TNFα
Smad6/7
Smurf1/2
Rbx1
Cul1
Skp1
THSD4
FBN1
THBS1
LTBP1
BAMBI
TGFβ RI
TGFβ RII
TGFβ
Decorin
FMOD
EMP3
+p
Smad2/3
SARA
Smad4
+u
+u
Ubiquitin mediated proteolysis
Transcription factors, co-activators, and co-repressors
Angiogenesis, extracellular matrix neogenesis, immunosuppression, apoptosis induction
Apoptosis
p107
E2F4/5
DP1
DNA
c-Myc
TGIF
p300
SP1
DNA
p15
G1 arrest
Cell Cycle
TAK1, MEKK1
DAXX/ JNK
MAPK signaling pathway
RhoA
ROCK1
PP2A
-p
p70S6K
IGSF1
BAMBI
Lefty
ActivinRI
ActivinRII
+p
Smad2/3
Smad6/7
Smad4
DNA
Gonadal growth, embryo differentiation, placenta formation, etc
FST
Activin
Nodal
NodalRI
NodalRII
+p
Smad2/3
Smad4
DNA
Pitx2
Left-right axis determination
Mesoderm and endoderm induction, etc
Data on KEGG graph
Rendered by Pathview

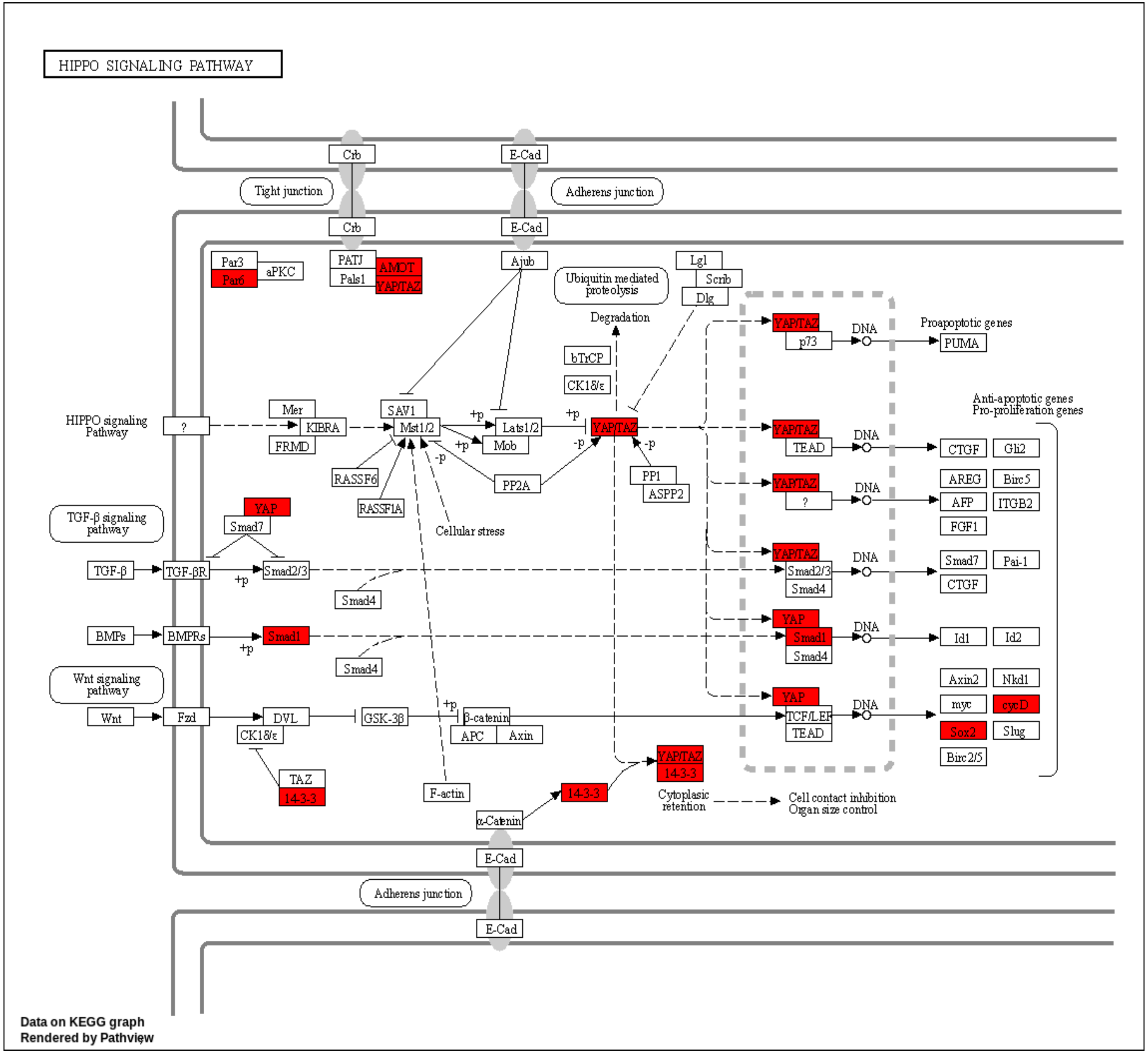
HIPPO SIGNALING PATHWAY
Crb
E-Cad
Tight junction
Adherens junction
Crb
E-Cad
Par3
Par6
aPKC
PATJ
Pals1
AMOT
YAP/TAZ
Ajub
Ubiquitin mediated proteolysis
Lgl
Scrib
Dlg
Degradation
bTrCP
CK1δ/ε
YAP/TAZ
p73
DNA
Proapoptotic genes
PUMA
Anti-apoptotic genes
Pro-proliferation genes
HIPPO signaling Pathway
?
Mer
KIBRA
FRMD
SAV1
Mst1/2
+p
Lats1/2
Mob
+p
-p
YAP/TAZ
-p
-p
PP1
ASPP2
RASSF6
RASSF1A
PP2A
Cellular stress
YAP/TAZ
TEAD
DNA
CTGF
Gli2
AREG
Birc5
YAP/TAZ
?
DNA
AFP
ITGB2
FGF1
TGF-β signaling pathway
YAP
Smad7
TGF-β
TGF-βR
+p
Smad2/3
Smad4
YAP/TAZ
Smad2/3
Smad4
DNA
Smad7
Pai-1
CTGF
BMPs
BMPRs
+p
Smad1
Smad4
YAP
Smad1
Smad4
DNA
Id1
Id2
Wnt signaling pathway
Wnt
Fzd
DVL
CK1δ/ε
GSK-3β
+p
β-catenin
APC
Axin
YAP
TCF/LEF
TEAD
DNA
Axin2
Nkd1
myc
cycD
Sox2
Slug
Birc2/5
TAZ
14-3-3
F-actin
α-Catenin
14-3-3
YAP/TAZ
14-3-3
Cytoplasic retention
Cell contact inhibition
Organ size control
E-Cad
Adherens junction
E-Cad
Data on KEGG graph
Rendered by Pathview

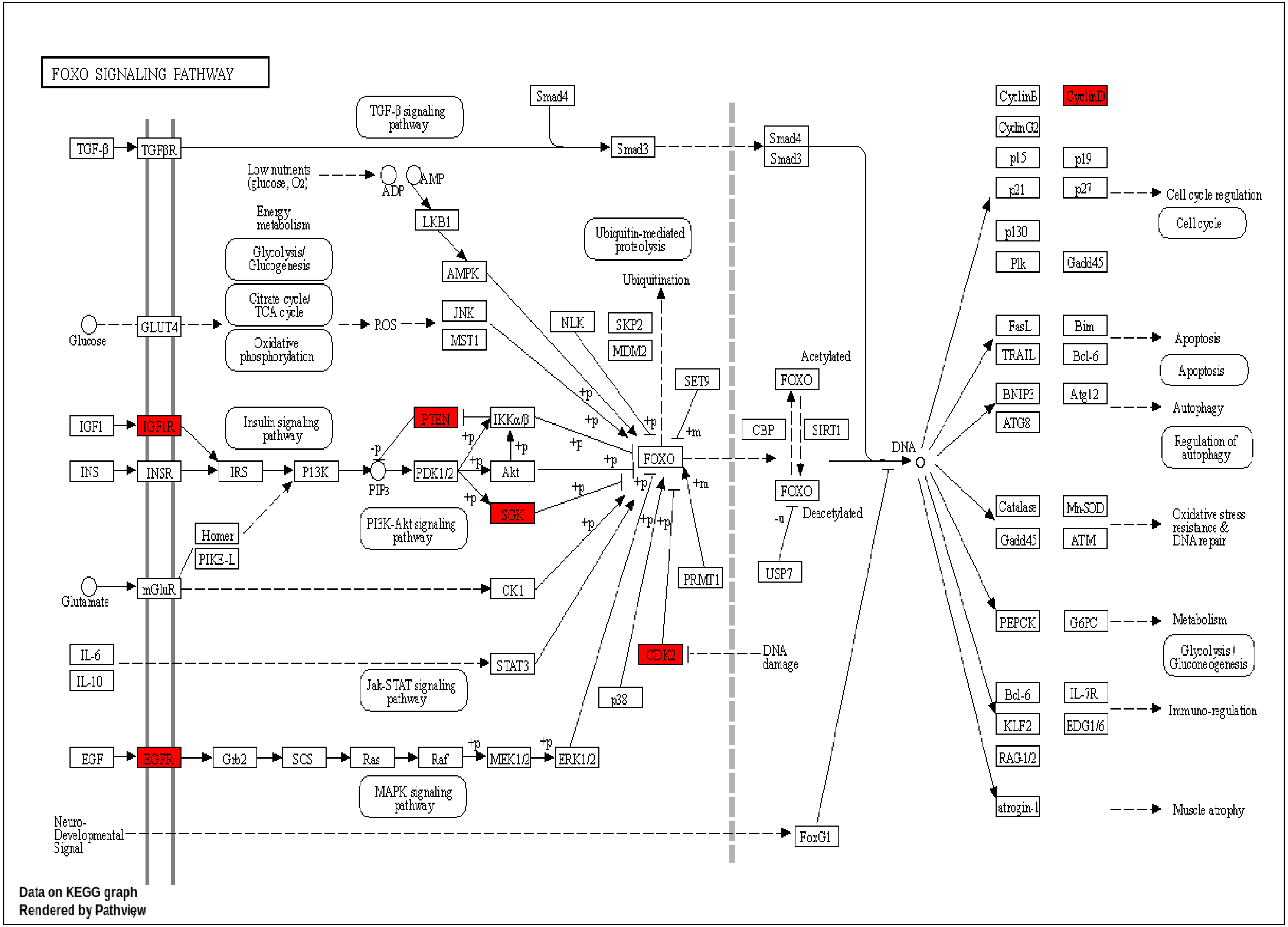
FOXO SIGNALING PATHWAY
TGF-β
TGFβR
TGF-β signaling pathway
Smad4
Smad3
Smad4
Smad3
Low nutrients (glucose, O2)
ADP
AMP
LKB1
Energy metabolism
Glycolysis/ Glucogenesis
Citrate cycle/ TCA cycle
Oxidative phosphorylation
AMPK
JNK
MST1
ROS
Glucose
GLUT4
Ubiquitin-mediated proteolysis
Ubiquitination
NLK
SKP2
MDM2
SET9
IGF1
IGF1R
Insulin signaling pathway
PTEN
IKKα/β
INS
INSR
IRS
PI3K
PIP3
PDK1/2
Akt
SGK
FOXO
PI3K-Akt signaling pathway
Homer
PIKE-L
Glutamate
mGluR
CK1
IL-6
IL-10
STAT3
Jak-STAT signaling pathway
CDK2
DNA damage
p38
PRMT1
EGF
EGFR
Grb2
SOS
Ras
Raf
MEK1/2
ERK1/2
MAPK signaling pathway
Neuro-Developmental Signal
FoxG1
Acetylated
FOXO
CBP
SIRT1
FOXO
Deacetylated
USP7
DNA
CyclinB
CyclinD
CyclinG2
p15
p19
p21
p27
Cell cycle regulation
Cell cycle
p130
Plk
Gadd45
FasL
Bim
TRAIL
Bcl-6
Apoptosis
Apoptosis
BNIP3
Atg12
ATG8
Autophagy
Regulation of autophagy
Catalase
MnSOD
Gadd45
ATM
Oxidative stress resistance & DNA repair
PEPCK
G6PC
Metabolism
Glycolysis / Gluconeogenesis
Bcl-6
IL-7R
KLF2
EDG1/6
Immuno-regulation
RAG1/2
atrogin-1
Muscle atrophy
Data on KEGG graph
Rendered by Pathview

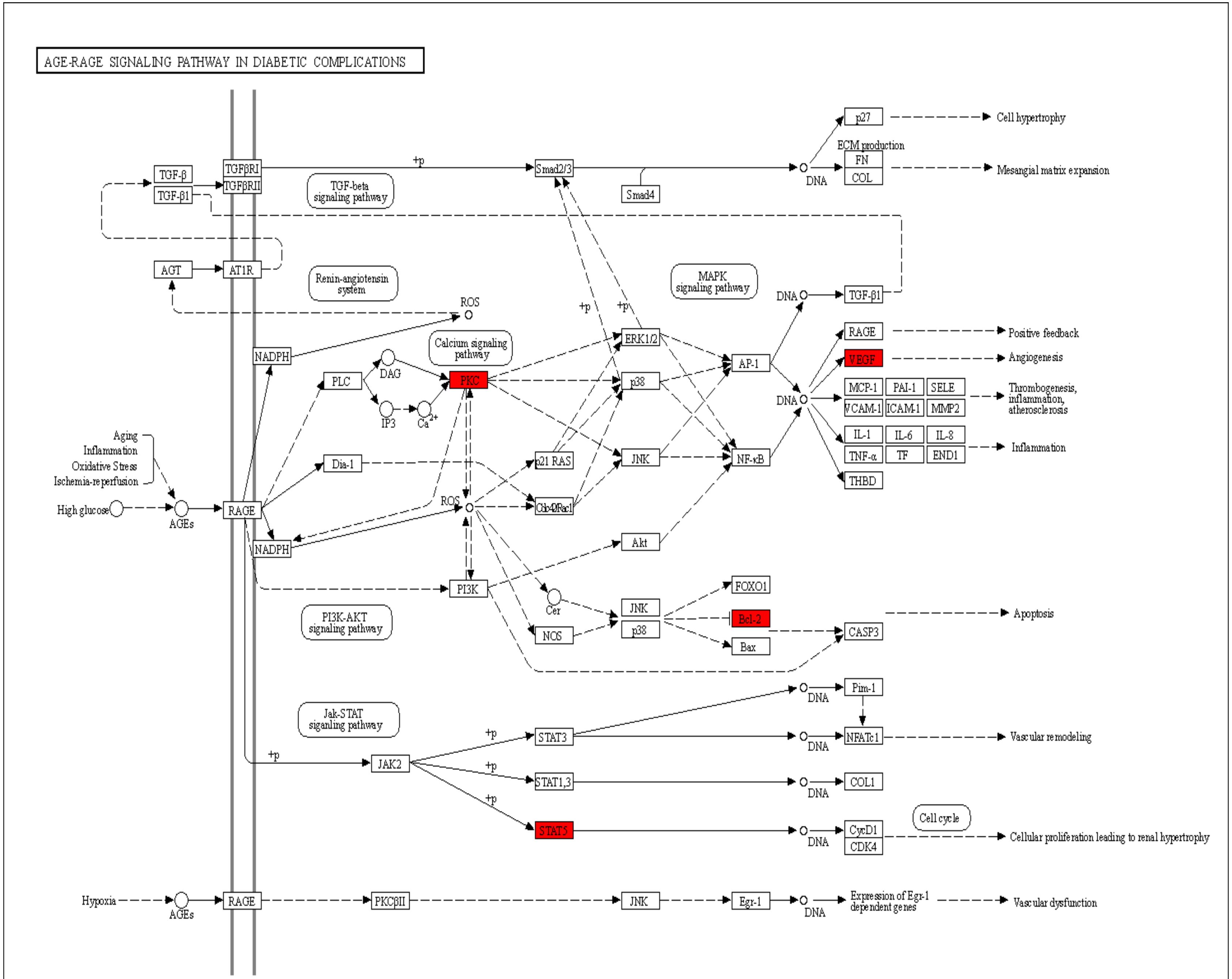

Data on KEGG graph
Rendered by Pathview

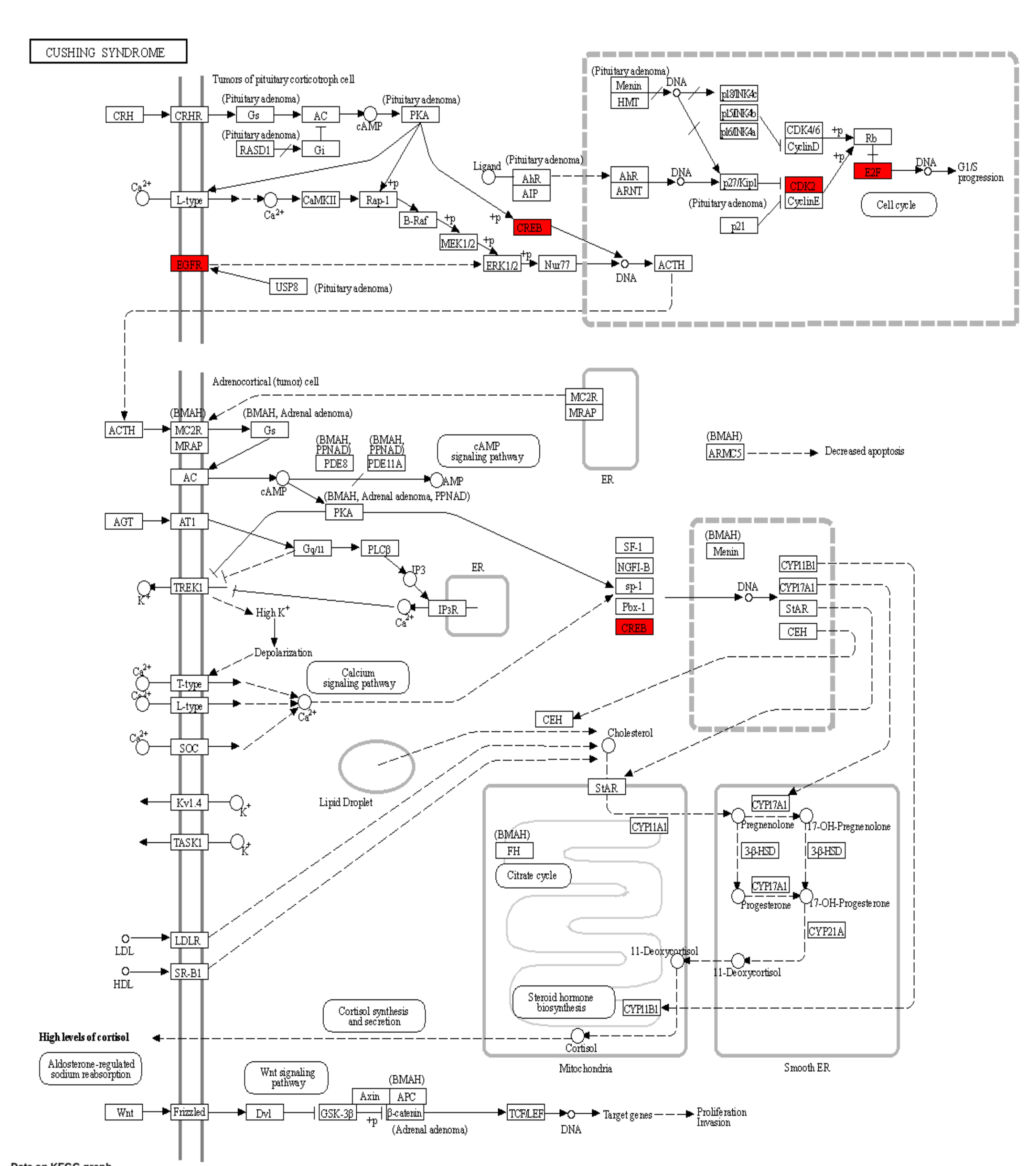

Data on KEGG graph
Rendered by Pathview

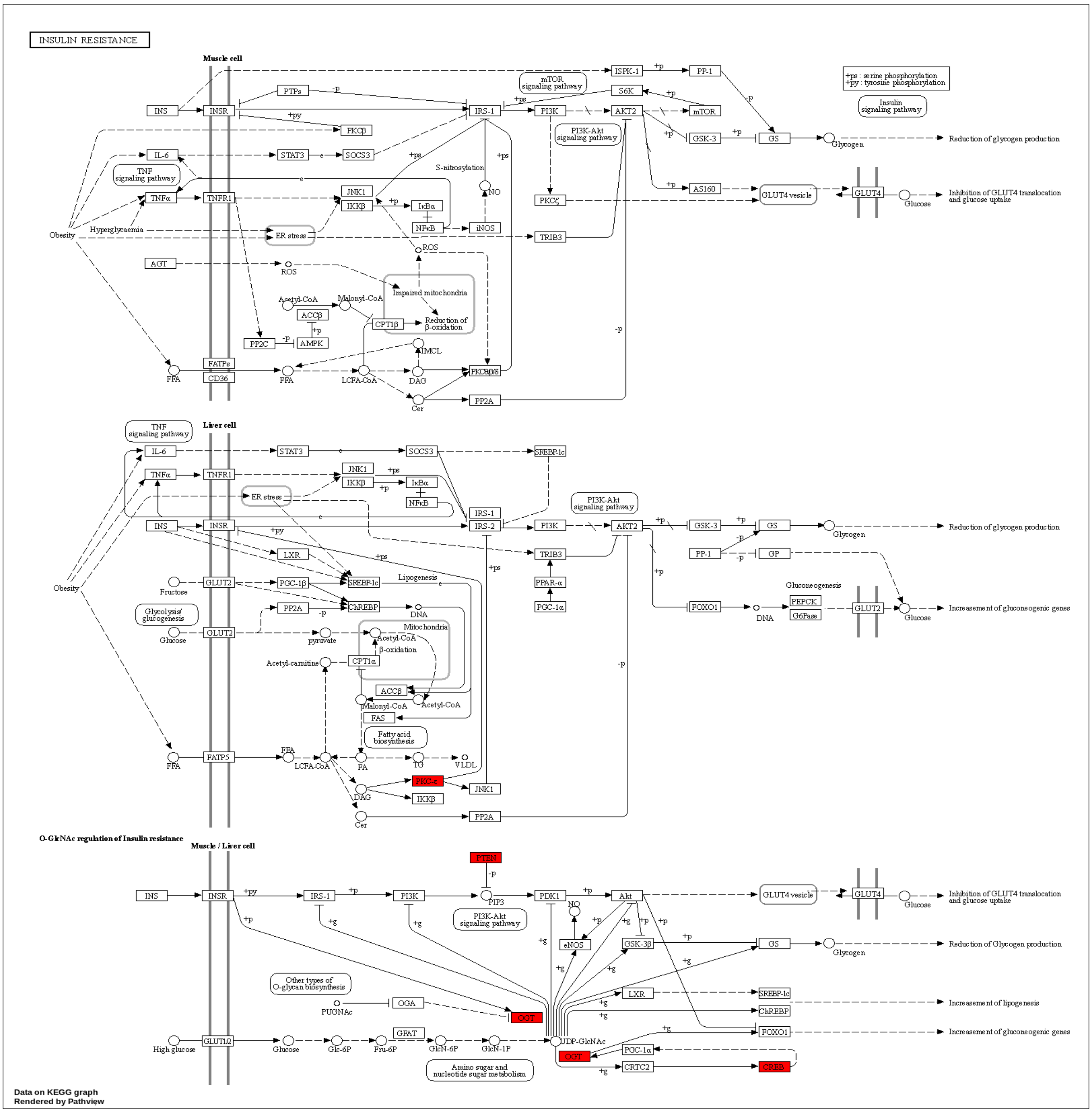
INSULIN RESISTANCE
Muscle cell
+ps : serine phosphorylation
+py : tyrosine phosphorylation
Insulin signaling pathway
mTOR signaling pathway
INS
INSR
PTPs
PKCβ
IRS-1
PI3K
AKT2
ISPK-1
PP-1
S6K
mTOR
GSK-3
GS
Glycogen
Reduction of glycogen production
IL-6
STAT3
SOCS3
TNF signaling pathway
TNFα
TNFR1
JNK1
IKKβ
IκBα
NFκB
iNOS
NO
S-nitrosylation
PI3K-Akt signaling pathway
PKCζ
AS160
GLUT4 vesicle
GLUT4
Glucose
Inhibition of GLUT4 translocation and glucose uptake
Obesity
Hyperglycaemia
ER stress
TRIB3
AGT
ROS
Acetyl-CoA
Malonyl-CoA
ACCβ
AMPK
PP2C
CPT1β
Impaired mitochondria
Reduction of β-oxidation
IMCL
FFA
FATPs
CD36
LCFA-CoA
DAG
PKCθ/β/δ
Cer
PP2A
Liver cell
SREBP-1c
IRS-2
LXR
GLUT2
Fructose
PGC-1β
Lipogenesis
ChREBP
DNA
Glycolysis/gluconeogenesis
pyruvate
Mitochondria
β-oxidation
Acetyl-carnitine
CPT1α
ACCβ
FAS
Fatty acid biosynthesis
FATP5
FA
TG
VLDL
PKC-ε
PPAR-α
PGC-1α
GP
FOXO1
Gluconeogenesis
PEPCK
G6Pase
Increasement of gluconeogenic genes
O-GlcNAc regulation of Insulin resistance
Muscle / Liver cell
PTEN
PIP3
PDK1
Akt
eNOS
GSK-3β
Reduction of Glycogen production
Other types of O-glycan biosynthesis
PUGNAc
OGA
OGT
SREBP-1c
Increasement of lipogenesis
High glucose
GLUT1/2
Glc-6P
Fru-6P
GFAT
GlcN-6P
GlcN-1P
UDP-GlcNAc
CRTC2
CREB
Amino sugar and nucleotide sugar metabolism
+p
-p
+py
+ps
+g
Data on KEGG graph
Rendered by Pathview

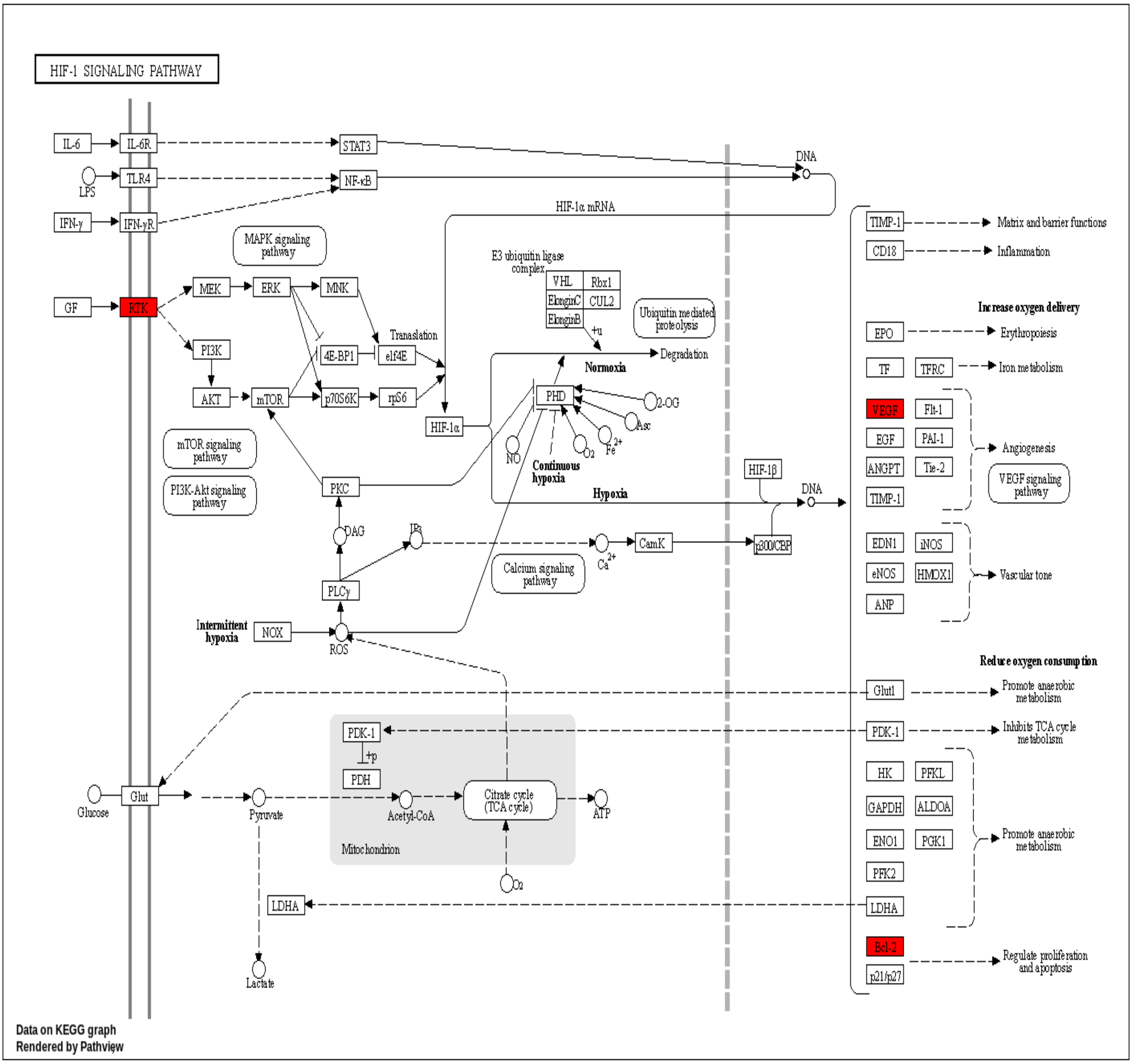
HIF-1 SIGNALING PATHWAY
IL-6
IL-6R
STAT3
LPS
TLR4
NF-κB
IFN-γ
IFN-γR
DNA
HIF-1α mRNA
MAPK signaling pathway
GF
RTK
MEK
ERK
MNK
PI3K
AKT
mTOR
4E-BP1
eIF4E
p70S6K
rpS6
Translation
HIF-1α
E3 ubiquitin ligase complex
VHL
Rbx1
ElonginC
CUL2
ElonginB
+u
Ubiquitin mediated proteolysis
Degradation
Normoxia
PHD
2-OG
Asc
O2
Fe2+
NO
Continuous hypoxia
Hypoxia
mTOR signaling pathway
PI3K-Akt signaling pathway
PKC
DAG
IP3
PLCγ
Ca2+
CamK
Calcium signaling pathway
HIF-1β
p300/CBP
Intermittent hypoxia
NOX
ROS
Glucose
Glut
Pyruvate
PDK-1
+p
PDH
Acetyl-CoA
Citrate cycle (TCA cycle)
ATP
Mitochondrion
O2
LDHA
Lactate
TIMP-1
Matrix and barrier functions
CD18
Inflammation
Increase oxygen delivery
EPO
Erythropoiesis
TF
TFRC
Iron metabolism
VEGF
Flt-1
EGF
PAI-1
ANGPT
Tie-2
TIMP-1
Angiogenesis
VEGF signaling pathway
EDN1
iNOS
eNOS
HMOX1
ANP
Vascular tone
Reduce oxygen consumption
Glut1
Promote anaerobic metabolism
PDK-1
Inhibits TCA cycle metabolism
HK
PFKL
GAPDH
ALDOA
ENO1
PGK1
PFK2
LDHA
Promote anaerobic metabolism
Bcl-2
p21/p27
Regulate proliferation and apoptosis
Data on KEGG graph
Rendered by Pathview

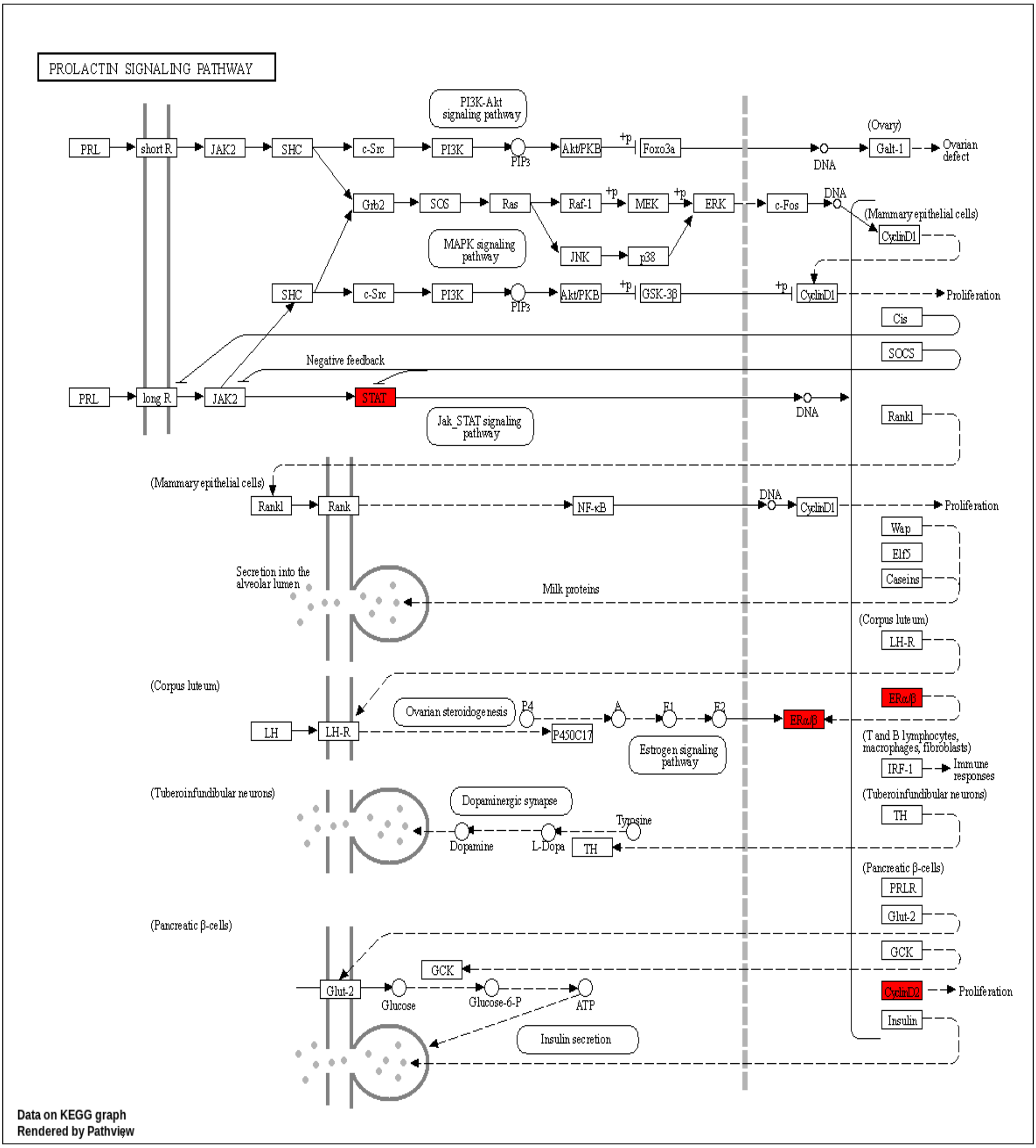
PROLACTIN SIGNALING PATHWAY
PI3K-Akt signaling pathway
PRL
short R
JAK2
SHC
c-Src
PI3K
PIP3
Akt/PKB
+p
Foxo3a
DNA
(Ovary)
Galt-1
Ovarian defect
Grb2
SOS
Ras
Raf-1
MEK
ERK
c-Fos
(Mammary epithelial cells)
CyclinD1
MAPK signaling pathway
JNK
p38
GSK-3β
Proliferation
Cis
SOCS
Negative feedback
long R
STAT
Jak_STAT signaling pathway
Rankl
Rank
NF-κB
Wap
Elf5
Caseins
Secretion into the alveolar lumen
Milk proteins
(Corpus luteum)
LH-R
LH
Ovarian steroidogenesis
P4
A
E1
E2
P450C17
ERα/β
Estrogen signaling pathway
(T and B lymphocytes, macrophages, fibroblasts)
IRF-1
Immune responses
(Tuberoinfundibular neurons)
Dopaminergic synapse
Tyrosine
Dopamine
L-Dopa
TH
(Pancreatic β-cells)
PRLR
Glut-2
GCK
Glucose
Glucose-6-P
ATP
CyclinD2
Insulin
Insulin secretion
Data on KEGG graph
Rendered by Pathview

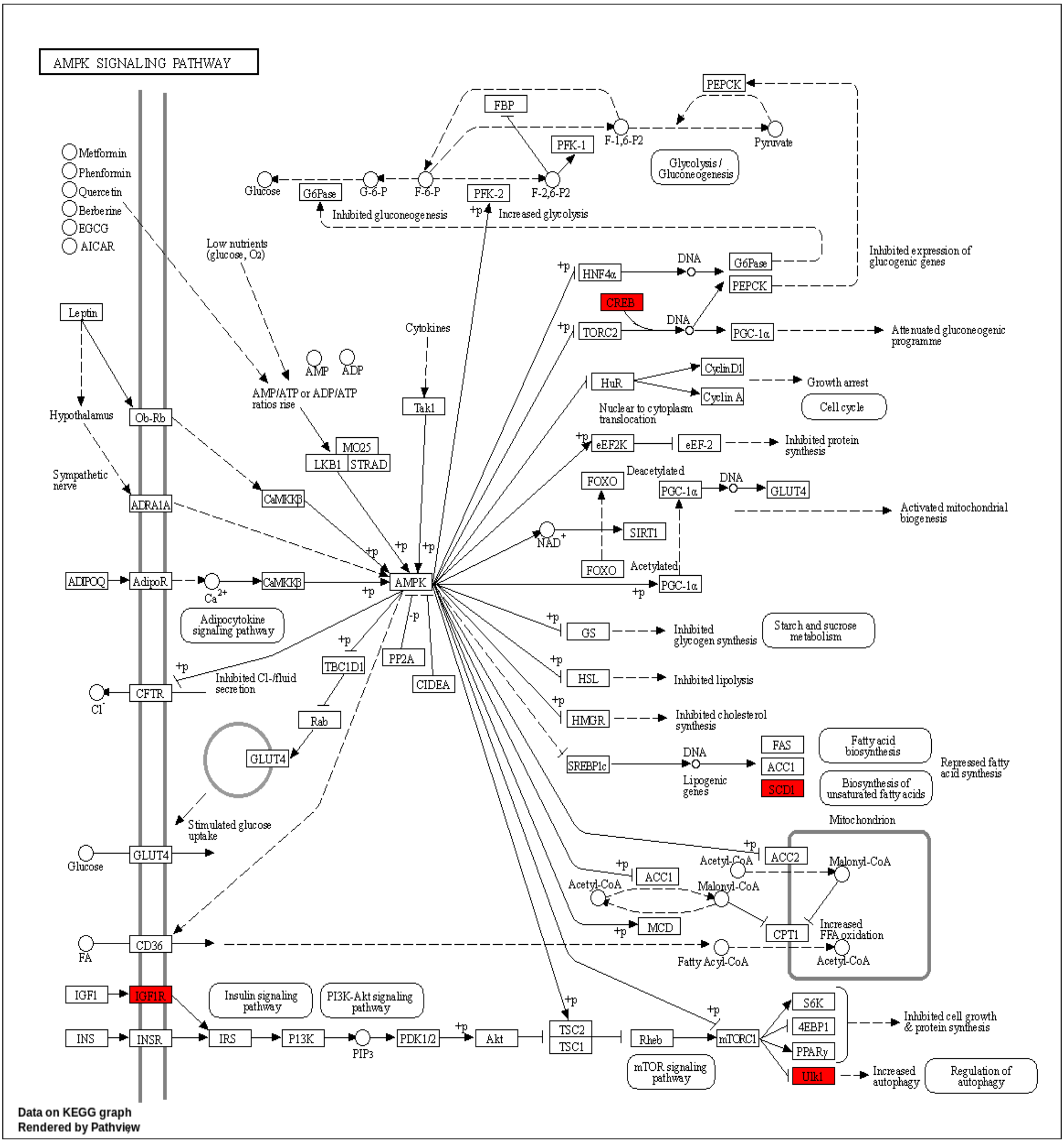

AMPK SIGNALING PATHWAY
Metformin
Phenformin
Quercetin
Berberine
EGCG
AICAR
Leptin
Hypothalamus
Ob-Rb
Sympathetic nerve
ADRA1A
ADIPOQ
AdipoR
CaMKKβ
Adipocytokine signaling pathway
CFTR
Inhibited Cl-/fluid secretion
GLUT4
Stimulated glucose uptake
Glucose
FA
CD36
IGF1
IGF1R
INS
INSR
IRS
P13K
PIP3
PDK1/2
Akt
Insulin signaling pathway
PI3K-Akt signaling pathway
TSC2
TSC1
Rheb
mTORC1
mTOR signaling pathway
S6K
4EBP1
PPARγ
Ulk1
Inhibited cell growth & protein synthesis
Increased autophagy
Regulation of autophagy
Low nutrients (glucose, O2)
AMP
ADP
AMP/ATP or ADP/ATP ratios rise
MO25
LKB1
STRAD
Cytokines
Tak1
AMPK
TBC1D1
Rab
PP2A
CIDEA
FBP
PFK-1
F-1,6-P2
Pyruvate
PEPCK
Glycolysis / Gluconeogenesis
Glucose
G6Pase
G-6-P
F-6-P
PFK-2
F-2,6-P2
Inhibited gluconeogenesis
Increased glycolysis
HNF4α
DNA
G6Pase
PEPCK
Inhibited expression of glucogenic genes
CREB
TORC2
PGC-1α
Attenuated gluconeogenic programme
HuR
CyclinD1
Cyclin A
Growth arrest
Cell cycle
Nuclear to cytoplasm translocation
eEF2K
eEF-2
Inhibited protein synthesis
FOXO
Deacetylated
GLUT4
NAD+
SIRT1
Acetylated
Activated mitochondrial biogenesis
GS
Inhibited glycogen synthesis
Starch and sucrose metabolism
HSL
Inhibited lipolysis
HMGR
Inhibited cholesterol synthesis
SREBP1c
Lipogenic genes
FAS
ACC1
SCD1
Fatty acid biosynthesis
Biosynthesis of unsaturated fatty acids
Repressed fatty acid synthesis
Mitochondrion
Acetyl-CoA
ACC2
Malonyl-CoA
MCD
CPT1
Increased FFA oxidation
Fatty Acyl-CoA
Data on KEGG graph
Rendered by Pathview
